# Progress on Transition Metal Ions Dissolution Suppression Strategies in Prussian Blue Analogs for Aqueous Sodium-/Potassium-Ion Batteries

**DOI:** 10.1007/s40820-024-01355-y

**Published:** 2024-02-21

**Authors:** Wenli Shu, Junxian Li, Guangwan Zhang, Jiashen Meng, Xuanpeng Wang, Liqiang Mai

**Affiliations:** 1 Department of Physical Science and Technology, School of Science, Wuhan University of Technology, Wuhan, 430070 People’s Republic of China; 2 School of Materials Science and Engineering, State Key Laboratory of Advanced Technology for Materials Synthesis, Wuhan University of Technology, Wuhan, 430070 People’s Republic of China; 3 Sanya Science and Education Innovation Park, Wuhan University of Technology, Sanya, 572000 People’s Republic of China; 4 Hubei Longzhong Laboratory, Wuhan University of Technology, Xiangyang Demonstration Zone, Xiangyang, 441000 People’s Republic of China

**Keywords:** Prussian blue analogs, Transition metal ions dissolution, Suppression strategies, Aqueous sodium-ion batteries, Aqueous potassium-ion batteries

## Abstract

Aqueous sodium-ion batteries (ASIBs) and aqueous potassium-ion batteries (APIBs) present significant potential for large-scale energy storage due to their cost-effectiveness, safety, and environmental compatibility. Nonetheless, the intricate energy storage mechanisms in aqueous electrolytes place stringent requirements on the host materials. Prussian blue analogs (PBAs), with their open three-dimensional framework and facile synthesis, stand out as leading candidates for aqueous energy storage. However, PBAs possess a swift capacity fade and limited cycle longevity, for their structural integrity is compromised by the pronounced dissolution of transition metal (TM) ions in the aqueous milieu. This manuscript provides an exhaustive review of the recent advancements concerning PBAs in ASIBs and APIBs. The dissolution mechanisms of TM ions in PBAs, informed by their structural attributes and redox processes, are thoroughly examined. Moreover, this study delves into innovative design tactics to alleviate the dissolution issue of TM ions. In conclusion, the paper consolidates various strategies for suppressing the dissolution of TM ions in PBAs and posits avenues for prospective exploration of high-safety aqueous sodium-/potassium-ion batteries.

## Highlights

- Comprehensive insights into Prussian blue analogs for aqueous sodium- and potassium-ion batteries.
- Unveiling the dissolution mechanism of transition metal ions in Prussian blue analogs.
- Innovative solutions to suppression transition metal ion dissolution, spanning electrolyte engineering, transition metal doping/substitution, minimize defects, and composite materials.

## Graphical abstract

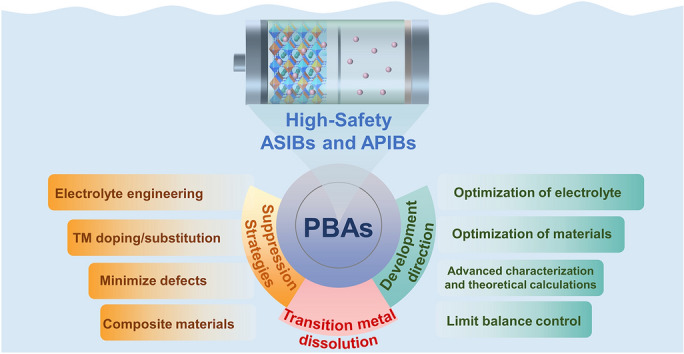

## Introduction

With the global economic and population growth, energy consumption has seen a pronounced rise, leading to accelerated depletion of fossil fuels and escalating environmental pollution. As a result, it becomes important to explore green renewable energy sources, such as wind, tidal, and solar energy. However, the intermittent nature and geographic limitations of these sources necessitate the development of extensive stationary energy storage systems (ESSs) to maximize their utility [1]. Presently, lithium-ion batteries (LIBs) are prevalent in the electric vehicle and portable electronic device segments, credited to their superior energy density and cycling stability [2, 3]. Yet, the limited availability and uneven distribution of lithium have driven up LIB costs, rendering them less suitable for the economical energy storage demands of ESSs [4]. Furthermore, the use of volatile and combustible organic electrolytes has been linked to battery-related fire incidents, compromising the ultra-high-safety requirements of ESSs [5–7]. Aqueous batteries, an emergent energy storage technology, are projected to offer a novel alternative for ESSs in the coming years [8–11].

**Fig. 1:**
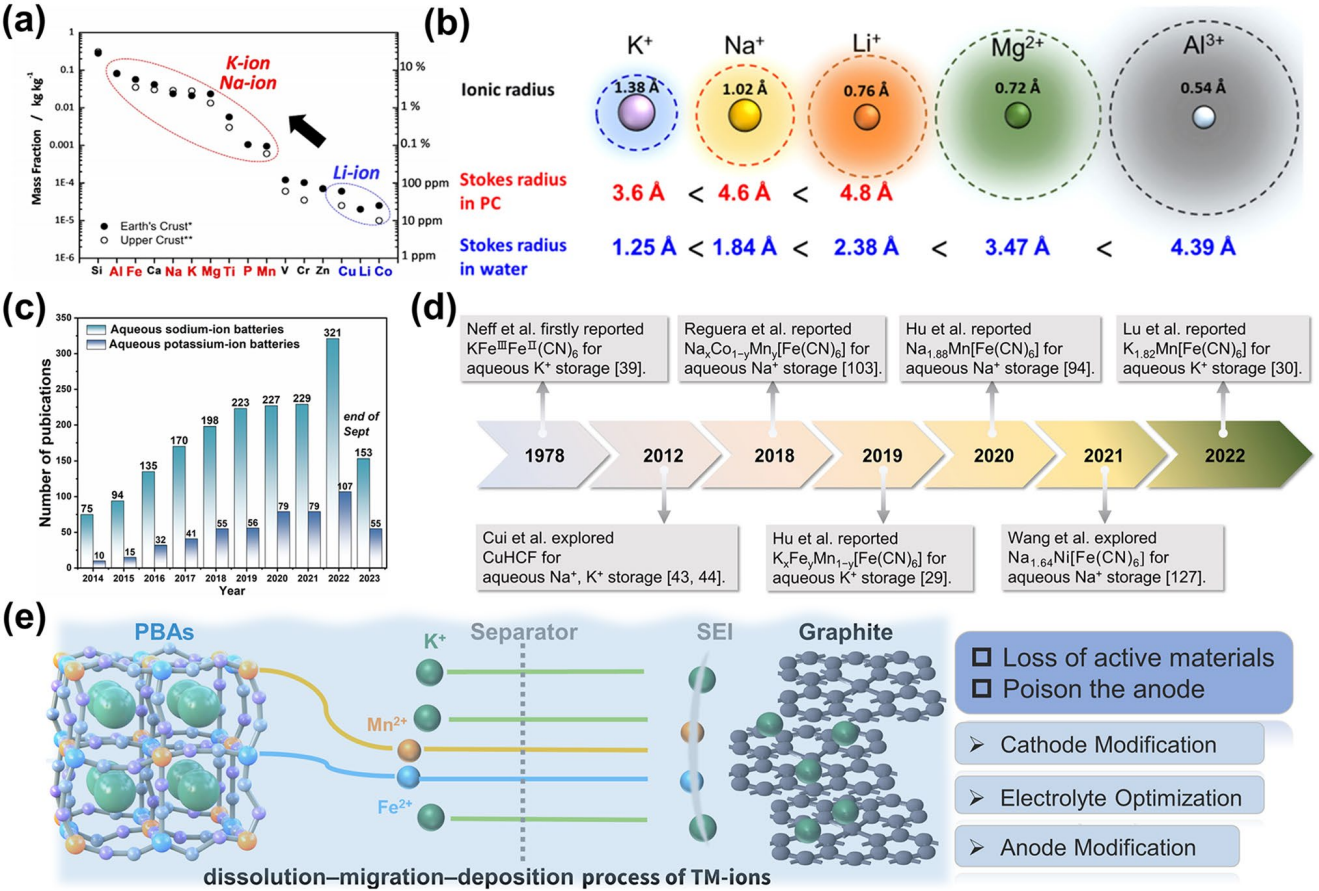
**a** The amount of sodium and potassium in the earth's crust compared to other elements [18]. Copyright 2018, Wiley–VCH. **b** The comparison of ionic radius and Stokes radius of Li^+^, Na^+^, K^+^, Mg^2+^, and Al^3+^ in water [19]. Copyright 2020, American Chemical Society. **c** The number of publications on ASIBs and APIBs over the past decade (as of Sept. 2023, generated by Web of Science using the keywords ‘Aqueous sodium-ion batteries’ and ‘Aqueous potassium-ion batteries’). **d** The development of PBAs as electrode materials for ASIBs and APIBs. **e** Schematic representation of Mn and Fe dissolution, migration, and deposition processes in aqueous batteries in PBA cathodes

Aqueous batteries offer superior safety, environmentally friendly qualities, and high ionic conductivity compared to non-aqueous LIBs, as a type of secondary battery that utilizes water as the electrolyte [12, 13]. Therefore, in pursuing more reliable and affordable energy storage solutions, aqueous batteries have emerged as a promising direction for current energy storage devices, with their improved safety, reliability, and affordability. For that reason, researchers are aggressively pushing on the development of aqueous batteries [14–16]. In 1994, Dahn et al. introduced the inaugural research on aqueous LIBs [17]. Within this novel category of aqueous rechargeable batteries, both aqueous sodium-ion batteries (ASIBs) and aqueous potassium-ion batteries (APIBs) have garnered significant interest, which is attributed to several prominent advantages. Firstly, they are cost-effective owing to the plentiful reserves of sodium and potassium combined with the elimination of strict assembly conditions and costly organic solvents, resulting in a marked reduction in the cost of ASIBs and APIBs (Fig. 1a) [18]. Secondly, they exhibit superior energy conversion efficiency. The Stokes radii of Na^+^ and K^+^ in water rank as the smallest among all ions (Fig. 1b) [19], facilitating rapid diffusion in the electrolyte. Additionally, the aqueous electrolyte delivers higher ionic conductivity than its organic counterpart, bestowing ASIBs and APIBs with remarkable power capability. Lastly, they promise enhanced safety, leveraging non-toxic, non-flammable, and eco-friendly aqueous solution electrolytes, which curtail risks during charge and discharge cycles [20]. As a consequence of these attributes, research concerning ASIBs and APIBs has witnessed an unprecedented surge over the past decade (Fig. 1c). Such batteries hold promise as optimal electrochemical tools for large-scale ESSs.

The advancement of efficacious electrode materials stands as a linchpin for the sustainable progress of ASIBs and APIBs. Such development hinges on melding exemplary electrochemical performance with the accessibility of low-cost materials. Hitherto, a multitude of electrode materials designed for ASIBs and APIBs has been methodically explored, encompassing layered metal oxides [21–23], polyanionic compounds [24–26], organic substrates [27–29], and Prussian blue analogs (PBAs) [30–32]. Notably, while metal oxides boast superior theoretical capacity, their synthesis methodology induces limited corrosion resistance to aqueous electrolytes and suboptimal cycling stability [33]. Anionic compounds, despite proffering elevated safety, cost-effectiveness, and environmental sustainability, grapple with capacity attrition due to dissolution in water. Organic materials, accentuated by apt operating voltages and pronounced reversible capacities, confront challenges stemming from diminished electronic conductivity. Conversely, PBAs distinguish themselves as a compelling electrode material for diverse battery systems, courtesy of their distinct open framework structure coupled with salient electrochemical attributes. Specifically, the three-dimensional (3D) open architecture avails expansive pore channels, accommodating the swift and reversible intercalation of guest cations. Moreover, this robust framework bestows commendable cycling stability, mitigating lattice volume fluctuations throughout the charge and discharge phases. Augmenting this, both the overarching structure and chemical makeup of PBAs can be judiciously modified to suit varied application requirements [34]. Remarkably, PBAs used in organic battery systems typically require consideration of the problem of crystalline water due to the potential for escaping lattice water during charging and discharging that can significantly damage the electrolyte and cause severe side reactions [35–39]. Conversely, PBAs in water-electrolyte environments rarely encounter such challenging concerns. The simplicity of their synthesis, anchored in a rudimentary water co-precipitation approach, paired with the overall modest raw material expenditure, earmarks them as auspicious candidates for broad-scale deployments [40]. Leveraging these virtues, PBA-based ASIBs and APIBs emerge as viable electrochemical energy storage apparatuses for expansive EESs.

In 1978, Neff et al. electrochemically synthesized PB (K_2_Fe^III^Fe^II^ (CN)_6_) thin-film electrodes using an aqueous solution comprising K_3_Fe(CN)_6_ and FeCl_3_ [41]. Evaluation of these PB electrodes in 1 M KCl aqueous solution for charge and discharge processes revealed cyclic voltammetry curves with a distinct pair of oxidation/reduction peaks. This suggested the reversible storage capability of K^+^ within PBs. Such foundational research on PBA thin-film electrodes, conducted prolifically between 1980 and 2000, set the trajectory for the eventual utilization of PBAs in aqueous batteries [42–44]. The dawn of the twenty-first century marked the onset of intensified research focusing on PBAs as electrode materials for aqueous batteries. In a notable instance from 2012, Cui et al. scrutinized the reversible insertion/extraction dynamics of Na^+^/K^+^ from PBA (CuHCF) electrodes in aqueous batteries [45, 46]. These CuHCF electrodes showcased remarkable attributes, including extended cycle longevity, multiplicative performance, superior round-trip energy efficiency, and cost-effective bulk synthesis. Such attributes render them promising for grid-scale energy storage applications. This seminal work catalyzed renewed enthusiasm toward PBAs within the realms of ASIBs and APIB systems. Subsequent endeavors by scholars have realized pivotal advancements in ASIBs and APIBs, principally via meticulous material design and electrolyte fine-tuning (Fig. 1d). Table 1 succinctly encapsulates details pertaining to electrolyte, discharge capacity, rate performance, and cycling statistics of emblematic PBA electrodes currently operational in ASIBs and PIBs.

**Table 1:** Electrochemical properties of the PBAs for ASIBs and APIBs

Work electrode	Counter electrode	Electrolyte	Discharge capacity (mAh g^−1^)	Rate capability (mAh g^−1^)	Cycling performance	References
*Aqueous sodium-ion batteries*						
Na_2_Mn[Fe(CN)_6_]	KMn[Cr(CN)_6_]	17 M NaClO_4_	40 at 1 C	25 at 30 C	N/A	[30]
Na_2_CoFe(CN)_6_	AC	1 M Na_2_SO_4_ + 1 wt% CoSO_4_	110.8 at 2 C	61 at 20 C	69.2%@10 C after 2000 cycles	[60]
Na_1.85_Co[Fe(CN)_6_]_0.99_·2.5H_2_O	Activated carbon (AC)	1 M Na_2_SO_4_	128 at 1 C	61 at 20 C	90%@5 C after 800 cycles	[61]
Na_1.24_Mn[Fe(CN)_6_]_0.8_·1.28H_2_O	NaTi_2_(PO_4_)_3_	17 M NaClO_4_	117 at 2.0 mA cm^−2^	56 at 20 mA cm^−2^	N/A	[93]
Na_2_MnFe(CN)_6_	NaTi_2_(PO_4_)_3_/C	32 M KAc and 8 M NaAc	57 at 0.1 A g^−1^	41 at 1 A g^−1^	N/A	[95]
Na_1.88_Mn[Fe(CN)6]_0.97_·1.35H_2_O	NaTiOPO_4_	9 M NaOTF + 22 M TEAOTF	41 at 0.25 C	N/A	76%@1 C after 800 cycles	[96]
Na_2_Mn[Fe(CN)_6_]	Pt	1 M Na_2_SO_4_ + 1 M ZnSO_4_ + SDS	137 at 0.5 C	100 at 30 C	74%@5 C after 2000 cycles	[97]
Na_1.58_Fe_0.07_Mn_0.97_Fe(CN)_6_ ·2.65H_2_O	PTCDI	17.6 M NaClO_4_ + 0.33 M Na_4_Fe(CN)_6_	157 at 0.5 A g^−1^	53 at 10 A g^−1^	73.4%@2 A g^−1^ after 15,000 cycles	[98]
[Ni(en)_2_]_3_[Fe(CN)_6_]_2_	Pt	0.5 M Na_2_SO_4_	36.5 at 0.1 A g^−1^	N/A	60.7%@1 A g^−1^ after 500 cycles	[100]
K_0.01_Cr_3_[Cr(CN)_6_]_2_⋅3.8H_2_O	MnFe PBA	17 M NaClO_4_	52.8 at 1 C	23 at 150 C	93.01%@30 C after 500 cycles	[101]
V/Fe PBA	Pt	0.5 M Na_2_SO_4_ + 5 M H_2_SO_4_	90 at 1.2 C	54 at 32 C	79%@16 C after 250 cycles	[102]
Na_1.65_Co_0.55_Mn_0.45_[Fe(CN)_6_]_0.87_	AC	1 M NaNO_3_	112.82 at 1 C	N/A	80%@1 C after 100 cycles	[105]
Na_2_Mn_0.2_Co_0.2_Ni_0.2_Cu_0.2_Zn_0.2_[Fe(CN)_6_]	NaTi_2_(PO_4_)_3_@C	1 M Na_2_SO_4_	75 at 0.5 C	57.1 at 10 C	87%@1 C after 5000 cycles	[109]
HQ-PB NCs	Pt	1 M Na_2_SO_4_	126.1 at 0.25 A g^−1^	83.3 at 3 A g^−1^	96.3%@1.25 A g^−1^ after 200 cycles	[115]
Na_1.17_Fe[Fe(CN)_6_]·0.35H_2_O	AC	17 M NaClO_4_	75 at 1 C	67 at 10 C	90%@10 C after 18,000 cycles	[123]
K_0.6_Ni_1.2_Fe(CN)_6_ ·3.6H_2_O	NiHCF	1 M NaNO_3_	59 at 0.83 C	40 at 41.7 C	100%@8.3 C after 5000 cycles	[127]
Na_1.64_Ni[Fe(CN)_6_]_0.89_·2.82H_2_O	5,7,12,14-Pentacenetetrone	17 M NaClO_4_	126 at 0.2 A g^−1^	87.9 at 50 A g^−1^	80%@2 A g^−1^ after 2000 cycles	[128]
Na_*x*_CuCNPFe	NaTi_2_(PO_4_)_3_@C@RGO	17 M NaClO_4_	45 at 0.025 A g^−1^	23 at 25 A g^−1^	N/A	[129]
NaMn_0.8_Fe_0.2_HCF	AC	2 M NaClO_4_ + 92PEG	100 at 0.1 A g^−1^	N/A	76%@0.2 A g^−1^ after 10,000 cycles	[130]
*Aqueous potassium-ion batteries*						
K_1.85_Fe_0.33_Mn_0.67_[Fe(CN)_6_]_0.98_·0.77H_2_O	PTCDI	22 M KCF_3_SO_3_	135 at 0.5 C	94 at 100 C	70%@100 C after 10,000 cycles	[31]
K_1.82_Mn[Fe(CN)_6_]_0.96_·0.47H_2_O	PTCDI	0.2 M Fe(CF_3_SO_3_)_3_ and 21 M KCF_3_SO_3_	160 at 0.3 A g^−1^	120 at 2.5 A g^−1^	99.9%@2.5 A g^−1^ after 130,000 cycles	[32]
CuHCF	AC/PPy	1 M potassium phosphate buffer	54 at 1 C	16.5 at 50 C	100%@10 C after 1000 cycles	[45]
K_0.71_Cu[Fe(CN) _6_]_0.72_·3.7H_2_O	CuHCF	1 M KNO_3_ + 0.01 M HNO_3_	59.14 at 0.83 C	40.1 at 83 C	83%@17 C after 40,000 cycles	[46]
K_2_NiFe(CN)_6_·1.2H_2_O	Graphite	1 M KNO_3_ + 0.01 M HNO_3_	77.4 at 5 C	42.1 at 500 C	98.6%@5 C after 5000 cycles	[69]
[Ni(en)_2_]_3_[Fe(CN)_6_]_2_	AC	0.5 M K_2_SO_4_	32.8 at 0.1 A g^−1^	30 at 5 A g^−1^	77.2%@0.5 A g^−1^ after 1000 cycles	[100]
CoFeHCF	AC	1 M KNO_3_	119 at 2 A g^−1^	N/A	51%@2 A g^−1^ after 4000 cycles	[103]
K_1.66_Fe_0.25_Co_0.75_[Fe(CN)_6_]·0.83H_2_O	AC	3 M KNO3	97 at 0.02 A g^−1^	43.4 at 1 A g^−1^	90.6%@0.2 A g^−1^ after 1000 cycles	[104]
K_2_Fe^II^[Fe^II^(CN)_6_] ·2H_2_O	Graphite	0.5 M K_2_SO_4_	120 at 1.4 C	104 at 14.4 C	85%@21.4 C after 500 cycles	[113]
K_1.93_Fe[Fe(CN)_6_]_0.97_ ·1.82H_2_O	AC	0.5 M Na_2_SO_4_ + 5 M H_2_SO_4_	142 at 0.075 A g^−1^	40 at 9 A g^−1^	88%@1.5 A g^−1^ after 300 cycles	[116]
Inverse opal PB single crystals	Pt	1 M KNO_3_	90 at 20 A g^−1^	N/A	100%@20 A g^−1^ after 1000 cycles	[117]
K_0.6_Ni_1.2_Fe(CN)_6_ ·3.6H_2_O	NiHCF	1 M KNO_3_	59 at 0.83 C	39 at 41.7 C	93%@8.3 C after 5000 cycles	[127]
K_3_[Fe(CN)_6_]/CNT	Pt	0.1 M KCl	75 at 0.2 A g^−1^	26 at 2 A g^−1^	N/A	[131]

ASIBs and APIBs share with numerous other aqueous batteries two fundamental constraints: constrained energy density and limited cycle life. These limitations primarily originate from the inherent hydrogen evolution reaction (HER) and oxygen evolution reaction (OER) in water, which constrict the electrochemical stability window, thereby undermining the batteries' energy density [47, 48]. As shown in Fig. 1e, Mn-based PBAs (e.g., MnHCF/graphite system) exhibited dissolution–migration–deposition (DMD) process of Mn and Fe. In PBA cathodes, water attacks transition metal ions with varying spin states in the structure, thus resulting in the dissolution of Mn^2+^ and Fe^2+^, loss of active material, and subsequent decreased capacity and structural stability. Electrolyte-dissolved ions have the potential to impede ion transport and disrupt the electrolyte environment. Additionally, Mn and Fe may accumulate on the anode, causing significant deterioration of the solid electrolyte interface (SEI) and hindering charge transfer. This phenomenon compromises the structural integrity of the electrode materials, subsequently impairing the battery's cycling stability.

Despite these challenges, a comprehensive compilation delineating the dissolution mechanisms of TM ions and corresponding mitigation tactics for PBAs in ASIBs and APIBs is conspicuously absent in contemporary literature. This mini-review addresses this gap, concentrating on the dissolution of TM ions in PBAs employed in ASIBs and APIBs and elucidating improvement strategies buttressed by seminal studies. Initially, an exploration of the structural attributes and redox processes of PBAs facilitates a thorough interpretation of the TM ions' dissolution mechanisms. Subsequent sections pivot to pragmatic formulation strategies to counter TM ions dissolution predicaments, encompassing electrolyte engineering, TM doping/substitution, minimize defects, and composite materials. Conclusively, this document recapitulates the tactics deployed to curtail TM ions dissolution in PBAs and postulates prospective research trajectories for high-safety aqueous sodium-/potassium-ion batteries.

## Mechanisms of TM Ions Dissolution in PBAs

### Structure and Redox Mechanism

**Fig. 2:**
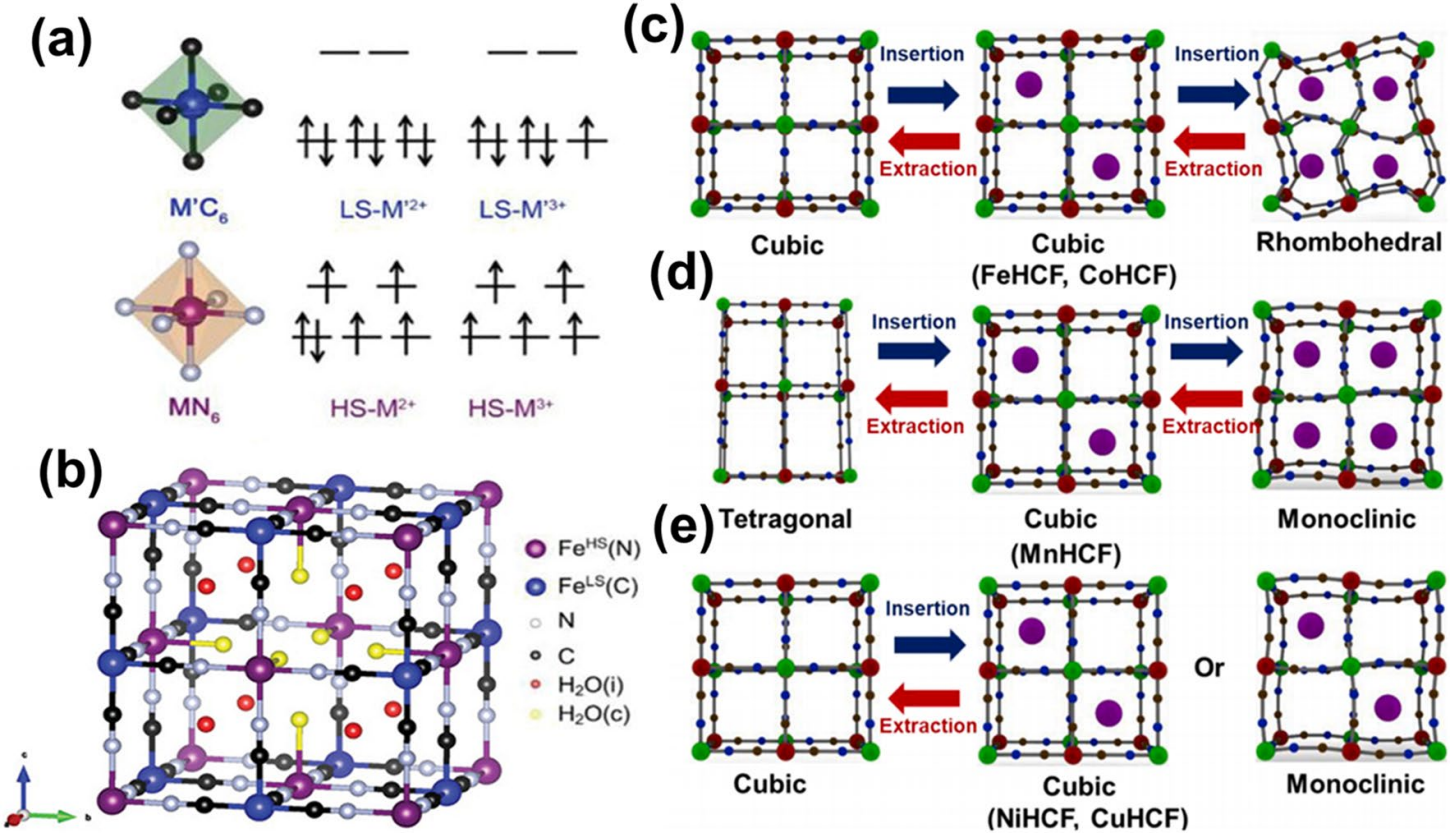
**a** Schematic illustration of the electronic states of TM ions in PBAs. **b** Schematic diagram of the general crystal structure of MHCF [50]. Copyright 2020, Wiley–VCH. Crystal structure evolution of **c** FeHCF and CoHCF, **d** MnHCF, and **e** CuHCF and NiHCF induced by insertion/extraction of AM ions during redox reactions [57]. Copyright 2022, Elsevier

PBAs possess a distinctive open metal–organic framework, categorizing them within a subset of coordination compounds characterized by the molecular formula A_*x*_M[M′(CN)_6_]_1-*y*_□_*y*_ ·zH_2_O (0 ≤ *x* ≤ 2, 0 ≤ *y* ≤ 1). Herein, 'A' signifies an alkali metal (AM) ion (such as Na^+^, K^+^), while 'M' and 'M′' represent TM ions, including but not limited to Fe, Co, Mn, Ni, and Cu. The symbol '□' denotes vacancies within the M′(CN)_6_ lattice, and 'H_2_O' refers to the water of crystallization, encompassing both interstitial and coordination water. Variables 'y' and 'z,' indicating the number of vacancies and water of crystallization, respectively, are contingent upon synthetic conditions, including the liquid-phase environment and the presence of chelating agents. Conversely, '*x*' is dictated by the cumulative valence of the TM ions [49]. Intriguingly, due to disparate 3D orbital splitting, the dual TM ions in PBAs manifest divergent spin states. As illustrated in Fig. 2a, coordination via C induces a more potent crystal field than coordination through N, engendering a broader partition in the M′ 3D orbitals compared to M. This differential results in M assuming a pronounced high spin (HS) state and M′ adopting a distinct low spin (LS) state [50–53].

The prevalent metal hexacyanoferrate (A_*x*_M[Fe(CN)_6_]_1-*y*_ □_*y*_·zH_2_O, denoted as MHCF) adopts a face-centered cubic (FCC) configuration anchored within a calcite-type framework. Within this FCC lattice, nitrogen and carbon atoms of the CN ligands engage with divalent or trivalent iron ions exhibiting varying spin states, thereby establishing Fe^HS^-N_6_ and Fe^LS^-C_6_ octahedra. These octahedral units are interconnected in an alternating sequence, culminating in the formation of expansive ion channels and substantial interstitial voids [38, 54]. Conventionally, PBAs are synthesized via aqueous co-precipitation, a methodology that inherently induces Fe(CN)_6_ vacancies and crystallization water within the structure, attributed to the unregulated, swift nature of the precipitation. To preserve electrostatic equilibrium, the structure incorporates a quarter of the Fe(CN)_6_ vacancies, derived from the Fe^3+^: Fe(CN)_6_ molar proportion (4:3), with a more illustrative representation of the crystal configuration presented in Fig. 2b. The omission of Fe(CN)_6_ sites prompts adjacent 6 HS Fe^3+^ to attain a state of unsaturation, compelling the ingress of six coordinating water molecules within these voids to sustain the cubic framework's integrity. It warrants emphasis that these vacancies exhibit a random distribution across the entire crystalline matrix. Additionally, each unit cell encompasses eight interstitial sites, each capable of accommodating a singular water molecule, culminating in 14 water molecules per unit cell [55]. In the context of nonaqueous batteries, the pronounced hydration level inherent to the crystalline structure predisposes the system to ancillary reactions, thereby elevating the significance of water expulsion as a pivotal impediment for PBA utilization within such battery systems. Contrarily, within ASIBs and APIBs, the crystallization water in PBAs demonstrates remarkable stability throughout charge–discharge cycles. MHCF's electrochemical processes, involving both the insertion/extraction of AM ions and the redox activities of M and Fe ions, proceed concomitantly. Notably, this charge–discharge protocol is explicable with reference to a structural model devoid of vacancies, excluding any participatory role of crystalline water in the reaction mechanism:

1 $$A_{x} M\left[ {Fe\left( {CN} \right)_{6} } \right]\cdot{\text{zH}}_{2} {\text{O }} \leftrightarrow {\text{ xA}}^{ + } + M\left[ {Fe\left( {CN} \right)_{6} } \right]^{ - x} \cdot{\text{zH}}_{2} {\text{O}}$$

During the charging phase, the sequential oxidation of M and Fe ions within MHCF facilitates the expulsion of AM ions from the crystalline lattice. Conversely, the discharge phase sees the reduction of M and Fe ions, concomitant with the reincorporation of AM ions. Should M represent redox-active elements such as Fe, Mn, or Co, the resulting FeHCF, MnHCF, and CoHCF display two sets of redox peaks, accommodating two AM ions and yielding a theoretical specific capacity of approximately 170 mAh g^–1^. However, this often incurs pronounced structural distortion and diminished cycling stability. In instances where M signifies an inert element, ZnHCF, NiHCF, and CuHCF proffer a solitary active Fe^2+^/Fe^3+^ redox site, attributable to the electrochemical inertness of Ni^2+^ and Cu^2+^, thereby constraining the specific capacity to a maximum of 80 mAh g^−1^, albeit typically accompanied by an exceptionally prolonged cycle life. Notably, the full activation of the Fe^LS^ redox reaction presents a substantial challenge due to the existence of Fe(CN)_6_ vacancies, culminating in an actual capacity for MHCF that falls short of theoretical projections. Concurrently, the redox process precipitates an evolution within the crystal structure, transitioning from cubic to monoclinic, rhombic, and, ultimately, tetragonal configurations. This structural metamorphosis predominantly hinges on the crystallization of water content alongside the specific types and concentrations of AM and TM ions [56, 57]. As evidenced in Fig. 2c, FeHCF and CoHCF predominantly maintain a cubic phase structure [58, 59]. The insertion/extraction of AM ions typically induces a bi-phase transition, oscillating between cubic and rhombic structures [60, 61]. An uptick in x within the lattice amplifies Coulombic interactions, prompting a systematic lattice expansion. Specifically, in the context of Na as the AM, the asymmetric distribution of electrons from TM ions across degenerate orbitals engenders divergent electron shielding effects in various directions for guest cations when x surpasses 1.5. This phenomenon triggers a transition from cubic to monoclinic configurations through structural distortion [61]. Subsequent diminution of crystallization water mitigates vesicular repulsion, distorting the crystal structure and facilitating the transition from monoclinic to rhombic phases [62]. Conversely, when K serves as the AM, configurations deficient in K typically exhibit a cubic structure [63], while K-enriched environments predominantly assume a monoclinic structural phase, impervious to the presence of crystallization water [64, 65]. Unlike FeHCF and CoHCF, MnHCF experiences intensified distortions, a consequence of structural aberrations invoked by the Jahn–Teller effect within the MnHS-N_6_ octahedral [66, 67]. Consequently, MnHCF undergoes a tri-phasic transformation spanning monoclinic, cubic, and tetragonal phases (Fig. 2d). In the case of NiHCF and CuHCF, characterized by the electrochemical inertness of Ni^2+^ and Cu^2+^, the cubic lattice undergoes minimal distortion, shifting marginally toward a monoclinic form or eschewing a structural phase transition altogether (Fig. 2e) [68, 69]. The repeated redox reactions always accompany the structural evolution, which leads to the dissolution of PBAs. NiHCF and CuHCF undergo minimal structural changes due to their characteristic of being zero-strain materials in long-term cycling, and their ultra-high structural stability makes TM ion dissolution more challenging. For the higher voltage plateau and dual redox centers increasing the capacity for FeHCF and CoHCF, the dissolution of TM ions becomes more accessible. Finally, the severe structural distortion caused by the Jahn–Teller effect of Mn leads to severe TM ions in MnHCF. Therefore, it is essential to comprehend the TM ion dissolution mechanism in PBAs to acquire more stable materials.

### TM Ions Dissolution

During charge and discharge processes, the cathode material predominantly employs TM ions as swift redox reaction centers to counterbalance the charge alterations accompanying the insertion and extraction of guest cations. The stability and electrochemical activity of TM ions critically influence the overall battery performance [70, 71]. Ideally, these TM ions should remain anchored within the cathode structure throughout the repeated cycles of cation insertion and extraction [72, 73]. Contrarily, in real-world scenarios, TM ions tend to dissociate from the cathode, diffuse into the electrolyte, and subsequently accumulate on the anode surface [74, 75]. This phenomenon contributes not only to the diminution of active materials and the alteration of the cathode's surface structure but also significantly affects the composition and electrochemical attributes of the solid electrolyte interface on the anode surface [76]. Consequently, even minimal quantities of TM ions involved in this DMD process can precipitate a substantial decline in both the capacity and power attributes of the battery. This issue stands as a paramount challenge for PBAs and constitutes one of the most substantial hurdles for transition metal-based cathode materials [77, 78].

**Fig. 3:**
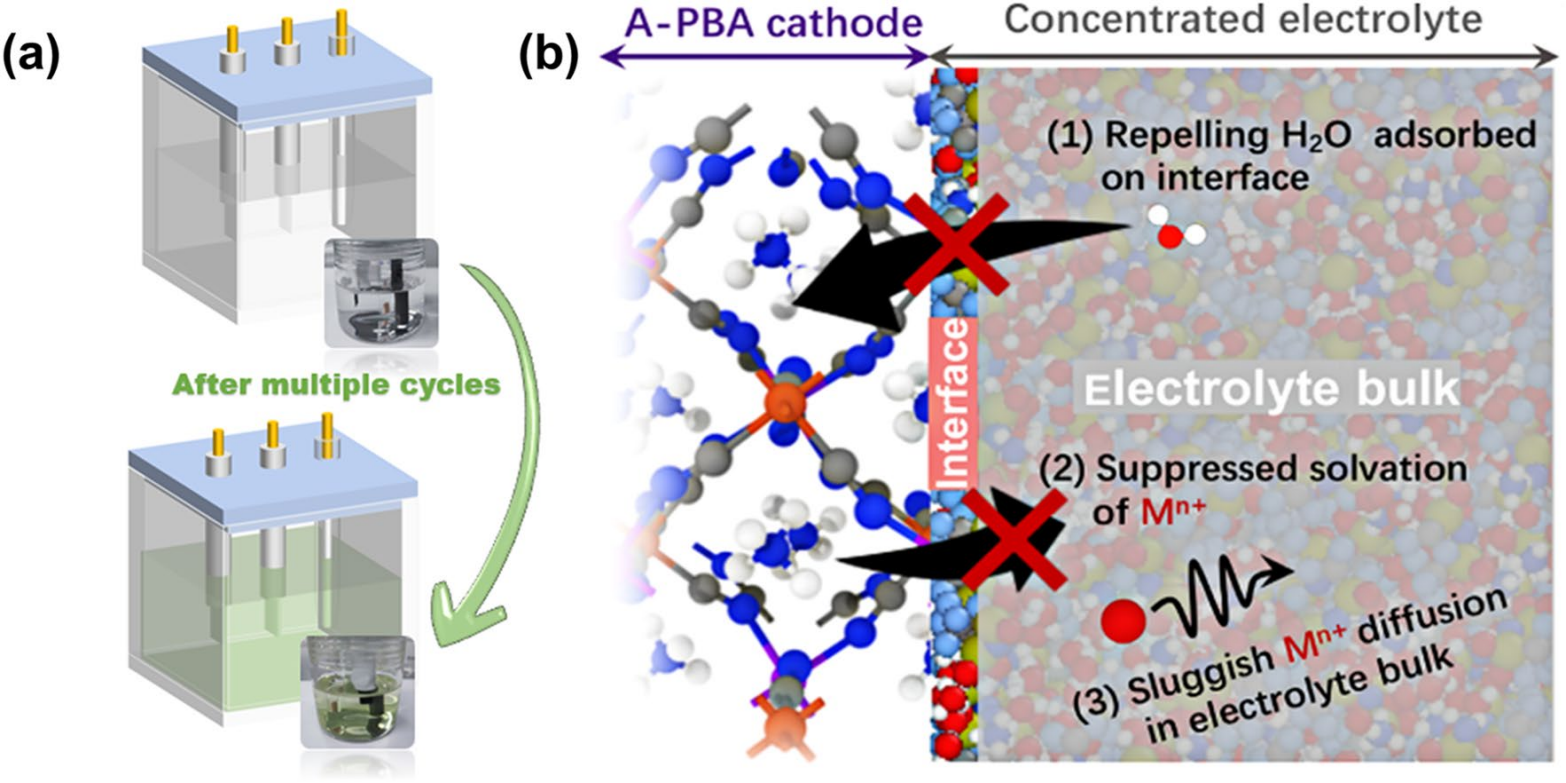
**a** Schematic representation of the dissolution phenomena in aqueous batteries of PBAs observable. The inset in the lower right corner shows the color change of the electrolyte after ten cycles of cycling at 0.3 A g^−1^ with 1 M KNO_3_ as the electrolyte, KMnHCF as the working electrode, and graphite as the counter electrode. **b** Schematic of critical factors in suppressing TM ions dissolution of PBA in the concentrated electrolyte [79]. Copyright 2022, American Chemical Society

The prevalent dissolution phenomenon is common in alkali-ion batteries (AIBs) with transition metal-based PBAs [77]. This intrinsic complication severely impairs the battery's capacity retention and cyclical stability [79]. Notably, a marked discoloration of PBA electrodes often occurs during the charge and discharge phases in aqueous solutions (Fig. 3a), attributed to the transition from insoluble to soluble forms of PBAs and exacerbated by intense water-induced dissolution of TM ions [80, 81]. Additionally, the incidental seepage of TM ions into the electrolyte is an unavoidable consequence of dynamic adsorption phenomena at the electrode interface [82]. Typically, the liberation of TM ions from PBAs encompasses three stages (Fig. 3b): Initially, a majority of TM ions migrate toward the surface of the PBA electrode. These ions undergo solvation by ambient water molecules at the PBA/electrolyte interface, followed by their diffusion into the aqueous electrolyte [83–85]. However, this dissolution and subsequent diffusion is significantly impeded in concentrated electrolytes. The peculiar network configuration of such electrolytes curtails the mobility of water molecules, resulting in sluggish diffusion kinetics. This, in turn, effectively deters TM ion dissolution, thereby enhancing the electrodes' cyclical stability within PBAs. Furthermore, PBAs containing Jahn–Teller active TM ions exhibit a pronounced rate of metal dissolution [86, 87]. Specifically, TM ions with Jahn–Teller activity experience augmented disproportionation in the presence of H^+^ in aqueous solutions. For instance, in MnHCF, higher valence Mn ions are readily reducible to Mn^3+^ or Mn^2+^ to offset Mn charge compensation. Concurrently, the renowned Jahn–Teller effect in Mn^3+^ also yields Mn^2+^ via Mn^3+^ disproportionation reactions [88]. Consequently, the Jahn–Teller effect induces a contraction of the four Mn-N bonds due to the [MnN_6_]^3+^ distortion, contrary to the stable [MnN_6_]^2+^, provoking unstable asymmetric contractions and expansions in PBA's octahedra throughout successive charge/discharge cycles. This instability facilitates Mn solubilization, culminating in progressive structural fracturing and severe battery capacity attrition [89]. Nevertheless, the multitude of variables inherent in PBA synthesis contributes to non-uniform defects. The crystallinity diverges post-polycrystalline assembly, complicating the straightforward quantification of dissolution kinetics. Hence, there is an urgent call for sophisticated characterization methodologies capable of accurately quantifying the dissolution of TM ions. Such advancements are pivotal for delving into the fundamental mechanisms governing the dissolution of TM ions and concomitant capacity deterioration, extending battery longevity [90].

## Strategies for Suppressing TM Ions Dissolution

**Fig. 4:**
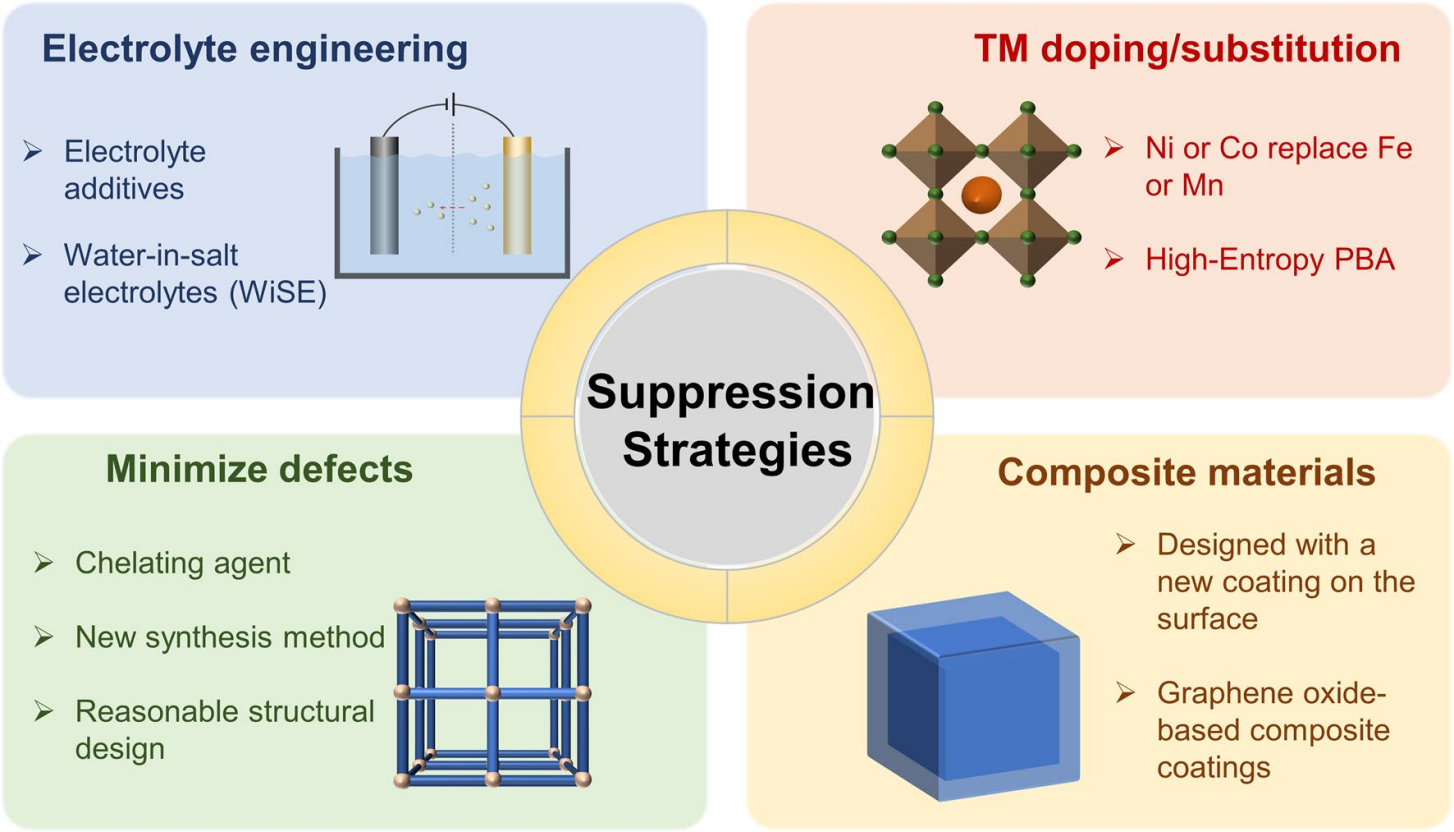
Strategies for suppressing TM ions dissolution in PBAs

The degradation of TM ions significantly impairs the integrity of PBAs, instigates stripping, diminishes capacity, and deteriorates the overall performance. Within both ASIBs and APIBs, the strategies of suppressing TM ions dissolution have been advanced to stabilize cycling life, as illustrated in Fig. 4. The suppression strategies can be comprised of four key aspects: electrolyte engineering, TM doping/substitution, defect minimization, and composite materials. Firstly, electrolyte engineering involves two main categories of electrolyte additives and highly concentrated electrolytes to safeguard the cathode against dissolution by reducing the impact of active water on it. Meanwhile, material synthesis and intrinsic structure perspectives are equally vital to impede dissolution. Secondly, TM doping or substitution can create a chemical composition and morphological structure for PBAs, adjusting the lattice parameters and redox properties, thus improving their structural stability. Thirdly, it is crucial to minimize the defects generated during the synthesis process after designing the appropriate chemical composition and morphological structure, as too many defects can irreversibly dissolve the materials and significantly damage their structural stability. Finally, further composite synthesis stabilizes the crystal structure and surface morphology. Notably, the surface coating creates a physical barrier layer that minimizes interfacial side reactions between the active material and electrolyte and inhibits TM ion dissolution.

Electrolyte engineering optimizes the external environment and the environment surrounding electrodes, which minimizing active water attack and reducing TM dissolution rate. The three latter strategies are synthetically designed to stabilize the structure, guaranteeing the solidity of the resulting PBA intrinsic structure. TM doping/substitution and defect minimization optimize the intrinsic structure and coat the obtained PBAs to enhance the surface structure and prevent material dissolution. TM doping/substitution and defect minimization optimize the intrinsic structure and coat the obtained PBAs to enhance the surface structure and prevent material dissolution. Based on the dynamic requirements of ESSs, it is imperative to address myriad issues caused by TM ions dissolution and necessitate innovative suppression strategies. Also, implementing multiple synergistic strategies is recommended to prevent the dissolution of TM ions, thereby accelerating the advancement of PBAs in ASIBs and APIBs.

### Electrolyte Engineering

In addressing the enhancement of energy density in ASIBs and APIBs, a critical obstacle remains the control of TM ion dissolution, necessitating a strategic focus on electrolyte engineering [91]. During the dissolution process, water molecules in the electrolyte are distinguished as either free or solvated water. Free water, exhibiting weak H–O bonds, is highly prone to deprotonation and hydrolysis, leading to spontaneous hydrolysis and subsequent by-product formation. Additionally, this reactive free water initiates the deterioration of the active material, resulting in substantial cathodic TM ion dissolution. Thus, the refinement of electrolyte engineering adheres to two central principles: the diminution of free water and the stabilization of dissolution equilibrium [92]. The water-in-salt electrolytes (WiSE) approach represents a paradigm for mitigating free water content, whereby various anions embed within the solvated structure and upon the electrode surface, impeding direct water–electrode material interaction. Nevertheless, when juxtaposed with nonaqueous cells, the constrained voltage window of aqueous electrolytes (~ 1.23 V) significantly curtails the potential output voltage and energy density of both ASIBs and APIBs. Okada et al. explored the ramifications of elevated electrolyte concentration on the operational voltage of ASIBs, employing Na_2_MnFe(CN)_6_ as the cathode and NaTi_2_(PO_4_)_3_ as the anode, with the objective of enhancing discharge voltage [93]. Cyclic voltammetry (CV) revealed that the electrochemical window with a high-concentration electrolyte (17 mol kg^–1^ NaCl) extended from 1.9 to 2.8 V, contrasting with its low-concentration (1 mol kg^–1^ NaCl) counterpart. The latter exhibited pronounced capacity decay following the initial cycle, attributed primarily to the anodic dissolution of TM ions provoked by the electrolytic reduction of water, yielding H_2_ and alkalinizing the electrolyte solution. Conversely, utilizing the extended stabilizing potential window of 17 mol kg^–1^ NaClO_4_, a discharge capacity of 117 mAh g^–1^ was achieved during the first cycle, alongside robust rate performance and cycling stability. Further examinations replaced 17 mol kg^–1^ NaClO_4_ with 9 mol kg^–1^ NaTFSI [30]. As NaF dissolves in saturated yet under-concentrated 9 mol kg^–1^ solution, the influx of OH^-^ and F^-^ ions from NaF compromises the structural integrity of PBAs, precipitating instability within alkaline solutions. Given the explosive and hygroscopic properties inherent to NaClO_4_, the exploration for more benign and stable salts is imperative. Subsequent studies by Ji et al. introduced a 30 M potassium acetate (KAc) electrolyte, achieving a broad 3.2-V electrochemical stabilization window and facilitating the reversible activity of anodic KTi_2_(PO_4_)_3_ in APIBs [94]. In a similar vein, Passerini et al. leveraged the high solubility of KAc, formulating a high-concentration cationic electrolyte with NaAc and pioneering the use of Al foil as the cathodic current collector in an aqueous milieu [95].

**Fig. 5:**
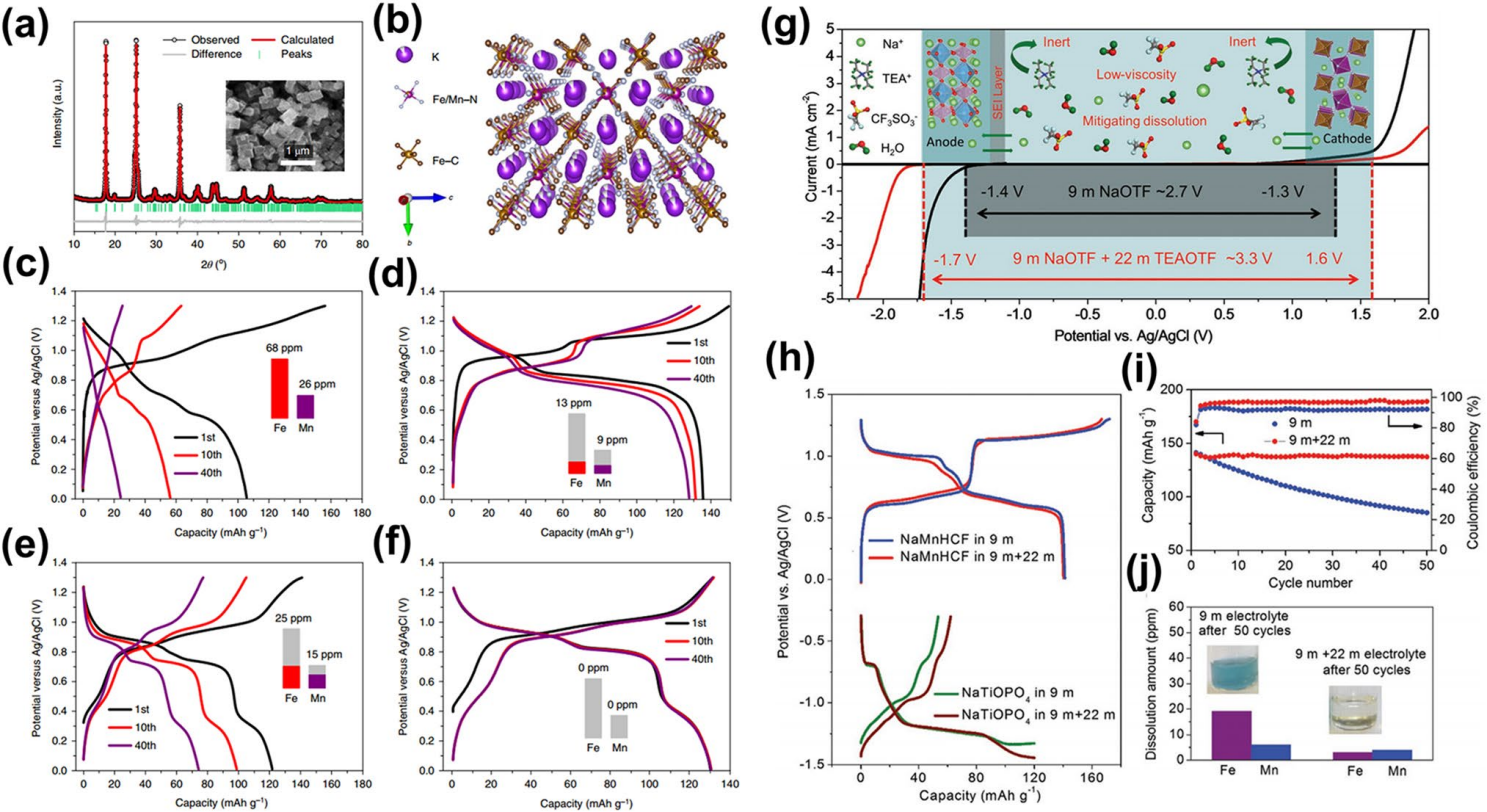
**a** XRD Rietveld refinement pattern for the KFeMnHCF-3565. **b** The typical structure of the K_*x*_Fe_*y*_Mn_1 −*y*_[Fe(CN)_6_]_*w*_·zH_2_O. Charge/discharge curves at 10 C: **c** KMnHCF and **d** KFeMnHCF-3565 in 1 M KCF_3_SO_3_ electrolyte, **e** KMnHCF and **f** KFeMnHCF-3565 in 22 M KCF_3_SO_3_ electrolyte [31]. Copyright 2019, Springer Nature. **g** Electrochemical voltage window of 9 mol kg^–1^ NaOTF and 9 mol kg^–1^ NaOTF + 22 mol kg^–1^ TEAOTF electrolytes. **h** Charge/discharge curves of the NaMnHCF cathode and NaTiOPO_4_ anode in 9 mol kg^–1^ NaOTF and 9 mol kg^–1^ NaOTF + 22 mol kg^–1^ TEAOTF electrolytes, respectively. **i** Long cycling of NaMnHCF electrodes in different electrolytes at 1 C. **j** The dissolving amount of the TM ions (Fe and Mn) after 50 cycles at 1 C [96]. Copyright 2020, Wiley–VCH

Despite the wide voltage window exhibited by KAc-based WiSE, its alkalinity (pH = 9) poses compatibility issues with PBAs. Hu et al. introduced a comprehensive APIB system featuring Fe-substituted PBA (KFeMnHCF-3565) cathode, 3,4,9,10-perylenetetracarboxylic diimide (PTCDI) anode, and 22 M KCF_3_SO_3_ WiSE electrolyte [31]. The X-ray diffraction (XRD) pattern and refinement results for KFeMnHCF-3565, depicted in Fig. 5a, confirm a monoclinic phase with a P21/n space group, complemented by a scanning electron microscope (SEM) inset displaying cubic structures measuring 200–800 nm per particle. Figure 5b illustrates a schematic of the quintessential open-framework crystal structure. Differing electrochemical behaviors under varying conditions are presented in Fig. 5c-f. The KMnHCF electrode, as revealed in Fig. 5c, underwent pronounced Fe and Mn dissolution during cycling in the electrolyte. A comparative assessment between Fig. 5c and d demonstrates that elevating the KMnHCF electrode's electrolyte concentration from 1 to 22 M enhanced capacity retention from 23 to 60%, marking a considerable improvement in cycling performance, yet it continued to experience capacity and voltage decline. In contrast, the KFeMnHCF-3565 electrode, characterized by partial Mn substitution with Fe, inhibited the dissolution of Fe and Mn to some extent, as evidenced in Fig. 5e and f, leading to enhanced capacity and cycling stability. Notably, the employment of 22 M KCF_3_SO_3_ concentrated electrolyte negated dissolution, capacity, or voltage degradation. In subsequent innovations, a unique WiSE for ASIBs was formulated by incorporating tetraethylammonium triflate (TEAOTF) salt (9 mol kg^–1^ NaOTF + 22 mol kg^–1^ TEAOTF), diminishing transition metal dissolution in the NaMnHCF cathode and markedly bolstering cycling performance [96]. Figure 5g underscores that 9 mol kg^–1^ NaOTF + 22 mol kg^–1^ TEAOTF offers an expanded electrochemical window (3.3 V), with the inset highlighting the electrolyte’s merits, including reduced viscosity and diminished electrode dissolution. The NaMnHCF cathode, delineated in Fig. 5h, manifested two voltage plateaus (0.7 and 1.0 V vs. Ag/AgCl). Remarkably, as Fig. 5i illustrates, the NaMnHCF electrode in 9 mol kg^–1^ NaOTF + 22 mol kg^–1^ TEAOTF electrolyte exhibited superior cycling stability, minimal capacity loss, and augmented average coulombic efficiency relative to the 9 mol kg^–1^ NaOTF electrolyte. Figure 5j reveals a substantial reduction in Fe and Mn dissolution in the 9 mol kg^–1^ NaOTF + 22 mol kg^–1^ TEAOTF electrolyte compared to its 9 mol kg^–1^ NaOTF counterpart. The Raman peak's broadening and diminution near 3487 cm^–1^ suggest that the mitigation of dissolution is attributed to the electrolyte's free water content, and the elevated electrolyte concentration impedes the free water’s corrosive effect on the electrode.

**Fig. 6:**
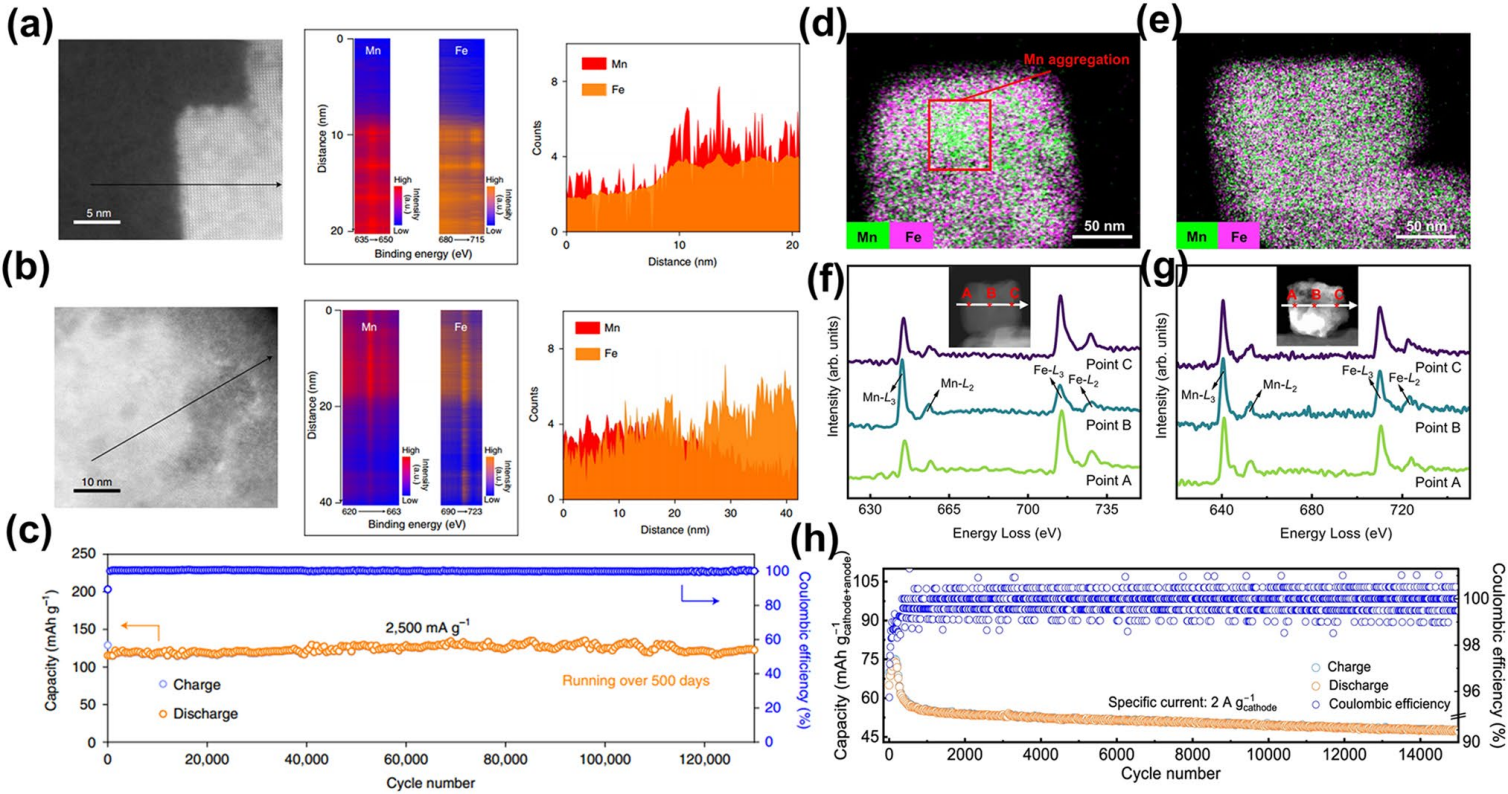
**a, b** The elemental content, intensity contour map, and image of the chosen cross-sectional area were acquired from the STEM-EELS line scan of the initial electrode and modified electrode. **c** Ultralong cycling stability of the KMnF in the modified electrolyte [32]. Copyright 2022, Springer Nature. **d, e** STEM mapping images of fully discharged electrodes in the blank and modified electrolyte. **f, g** EELS line scans of fully discharged electrodes for 300 cycles of PTCDI||NaFeMnF in blank and modified electrolytes, respectively, at 0.5 A g^−1^. **h** Cycling performance of the PTCDI||NaFeMnF at 2 A g^−1^ [98]. Copyright 2023, Springer Nature

The utilization of economical electrolyte additives presents a more pragmatic and feasible approach compared to the high salt requisites of WiSE. Qian et al. have demonstrated that the introduction of the surfactant sodium dodecyl sulfate (SDS) into the aqueous medium effectively mitigates hydrogen or oxygen evolution at the electrode interface, thereby extending the electrochemical stability window of the electrolyte to approximately 2.5 V [97]. In this context, SDS molecules adhere to the electrode surface via electrostatic interactions, effectively precluding the precipitation of hydrogen or oxygen. Employing Na_2_MnFe(CN)_6_ as the cathode and zinc sheets as the anode, the resulting aqueous rechargeable hybrid ion battery manifests an energy density peaking at 170 Wh kg^–1^, maintaining capacity retention of 75% across 2000 cycles within an operational voltage of 2.0 V. Energy density computations, grounded in density flooding theory, underscore the pivotal role of SDS in curbing water splitting, manganese dissolution, and anode degradation, thereby enhancing cyclic durability and rate capacity. Empirical evidence suggests that the incorporation of surfactants into aqueous electrolytes augments the electrochemical stability window to 2.5 V. Adjusting the electrolyte composition to match the TM ions of the cathode alters dissolution equilibria, curbing continuous active material attrition, and the incorporation of TM ions within the cathode serves to fortify the structural integrity. Furthering this approach, Li et al. succeeded in bolstering the structural stability of Na_2_CoFe(CN)_6_ through the integration of specific electrolyte additives (1 wt% CoSO_4_) within an aqueous 1 M Na_2_SO_4_ electrolyte [60]. The structural alterations of the electrode skeleton throughout the cyclic process have been examined via XRD, Fourier transform infrared spectroscopy (FTIR), and inductively coupled plasma atomic emission spectroscopy (ICP-AES). These analyses have elucidated that the presence of Co^2+^ ions in the electrolyte effectively precludes both the disintegration of the CoHCF skeletal framework and the solubilization of TM ions within the structure during cycling. Similarly, Lu et al. instigated an in situ electrochemical cation substitution in the KMnF cathode by incorporating 0.2 M Fe(CF_3_SO_3_)_3_ into a 21 M KCF_3_SO_3_ electrolyte [32]. This innovative approach to surface modification markedly curtailed manganese dissolution, thereby enhancing both structural and surface chemical stabilities of the electrode. Investigations involving scanning transmission electron microscopy (STEM) electron energy loss spectroscopy line scans and elemental mapping were executed on the cross-sectional regions of both original and altered electrodes. Figure 6a and b displays intensity contour plots and elemental compositions, demonstrating a uniform presence of Fe and Mn in both initial and modified electrodes. Notably, the surface of the altered electrode showed a substantial relative increase in Fe in contrast with Mn, solidifying the conclusion that Fe^3+^ is integrated into the electrode framework during cycling in the amended electrolyte. Comprehensive characterizations indicated that Fe present in the modified electrolyte compensates for the Mn vacancies generated in situ within the KMnF structure. Consequently, the KMnF electrode revealed exceptionally prolonged cyclical stability, sustaining a capacity of roughly 120 mAh g^–1^ following 130,000 cycles at 2500 mA g^–1^ without notable capacity attrition per cycle (Fig. 6c). In a more nuanced approach, Wang and associates ingeniously incorporated Na_4_Fe(CN)_6_ as a supplemental salt in the concentrated aqueous NaClO_4_-based electrolyte solution, aiming to occupy the Mn vacancies emergent on the surface [98]. Addressing the lattice expansion ensuing from Na^+^ extraction, the [Fe(CN)_6_]^4−^ anion was employed to engage with dislocated or displaced Mn, thereby rectifying Mn vacancies in situ and fortifying the surface's chemical stability. Post 300 cycles utilizing diverse electrolytes, Fig. 6d illustrated pronounced Mn accumulation within individual particles when subjected to the standard electrolyte. Conversely, Mn distribution remained uniform across particles in the presence of the modified electrolyte (Fig. 6e), denoting the efficacious curtailment of Mn dissolution. Subsequent analyses employed electron energy-loss spectroscopy (EELS) on the particles' cross-sections harvested from the cycling electrode (Fig. 6f and g). Within the unmodified electrolyte system, the Mn L_3_/L_2_ intensity ratio surged markedly from 4.02 (point A) to 5.18 (point B). This alteration in ratio underscored the Mn's inhomogeneous valence state within discrete particles and the persistence of an elevated valence state, potentially linked to amorphous compounds emanating from structural degradation. In stark contrast, the Mn L_3_/L_2_ intensity ratio within the modified electrolyte system exhibited negligible fluctuation, signifying a consistent valence state of Mn across the particles. As delineated in Fig. 6c, the capacity retention of the PTCDI||NaFeMnF stood at 73.4% following 15,000 cycles at 2 A g^–1^, with the specific capacity sustained at 47.5 mAh g^–1^, and the coulombic efficiency approached a near-perfect 100%.

WiSE, the highly concentrated electrolytes, has been proven effective in expanding the electrochemical stabilization window of aqueous electrolytes and improving low-temperature performance through increased salt concentration. However, its practical applications could be limited for its high viscosity, low conductivity, and high cost. In particular, their increased cost and viscosity do not offer enough benefits. The incorporation of organic solvents, such as dimethyl carbonate and acetonitrile, can effectively decrease the viscosity of WiSE while also decreasing salt content and preventing water proton activity [99]. However, due to their toxicity and flammability, organic additives are unsuitable for use as electrolyte additives in aqueous solutions.

By effective strategies incorporating electrolyte additives and WiSE, electrolyte engineering has aroused great concern and demonstrates the ability to mitigate TM ion dissolution. Ultra-high salt concentrations decrease active water content and increase viscosity, thereby hindering the dissolution and diffusion of substances on electrode surfaces. Heightened viscosity and slower interfacial processes contribute to reducing overall rate performance. However, the cost implications of salt concentration cannot be ignored as high-energy density and long cycle life are undoubtedly the most critical features for a large-scale energy storage device. Additionally, incorporating cation trapping agents into the electrolyte enables in situ repair of surface defects formed during charging and discharging, improves dissolution equilibrium, and further enhances cycling stability.

### TM Doping/Substitution

The process of TM doping/substitution involves the replacement of certain or all sites within the original matrix with alternative ions or groups, representing a paramount strategy in the material optimization studies of PBAs. This technique engenders structural vacancies, occupies voids, or meticulously adjusts lattice parameters, contributing to the enhancement of structure and inherent properties. PBAs, predicated on redox behavior, are categorized into two primary types: dual-electron transfer PBAs (DE-PBAs, with M and M′ encompassing elements such as Mn, Fe, and Co) and single-electron transfer PBAs (SE-PBAs, where M includes Cu, Ni and M′ involves Fe, Co, and Mn). DE-PBAs, despite possessing a superior theoretical capacity, often undergo substantial structural degradation due to significant deformation experienced during charge and discharge cycles. Conversely, SE-PBAs exhibit commendable cycling stability and elevated electrical conductivity but are constrained by a lower capacity attributable to the presence of a single active redox component. Consequently, the role of doping/substitution becomes indispensable in augmenting their electrochemical attributes, encompassing aspects such as specific capacity, rate capability, and operational potential.

Zhao et al. reported the synthesis of potassium-rich mesoporous K_2_NiFe(CN)_6_·1.2H_2_O, utilizing Ni in lieu of FeHCF, serving as the cathode for ultrafast APIBs [69]. This innovative substitution strategy propelled APIBs to reach a groundbreaking rate capability of up to 500 C (8214 W kg^–1^), completing a single charge/discharge cycle in a mere 4.1 s. In a distinct approach, Yan et al. replaced the Ni site in NiHCF with a [Ni(en)_2_]^2+^ moiety, culminating in an entirely novel structure, [Ni(en)_2_]_3_[Fe(CN)_6_]_2_ (NienHCF), which was employed for the first time as a cathode material in ASIBs and APIBs [100]. The APIBs exhibited robust cycling stability, maintaining 77.2% of their initial capacity after 1000 cycles at 0.5 A g^–1^, in conjunction with an activated carbon anode. In another development, Chen et al. detailed that Cr-substituted CrCr-PBA displayed enhanced performance in ASIBs [101]. The incorporation of Cr contributed two redox pairs at potentials of 0.62/0.65 V and 0.73/0.77 V, respectively, coordinating at two different sites mediated by C and N.

**Fig. 7:**
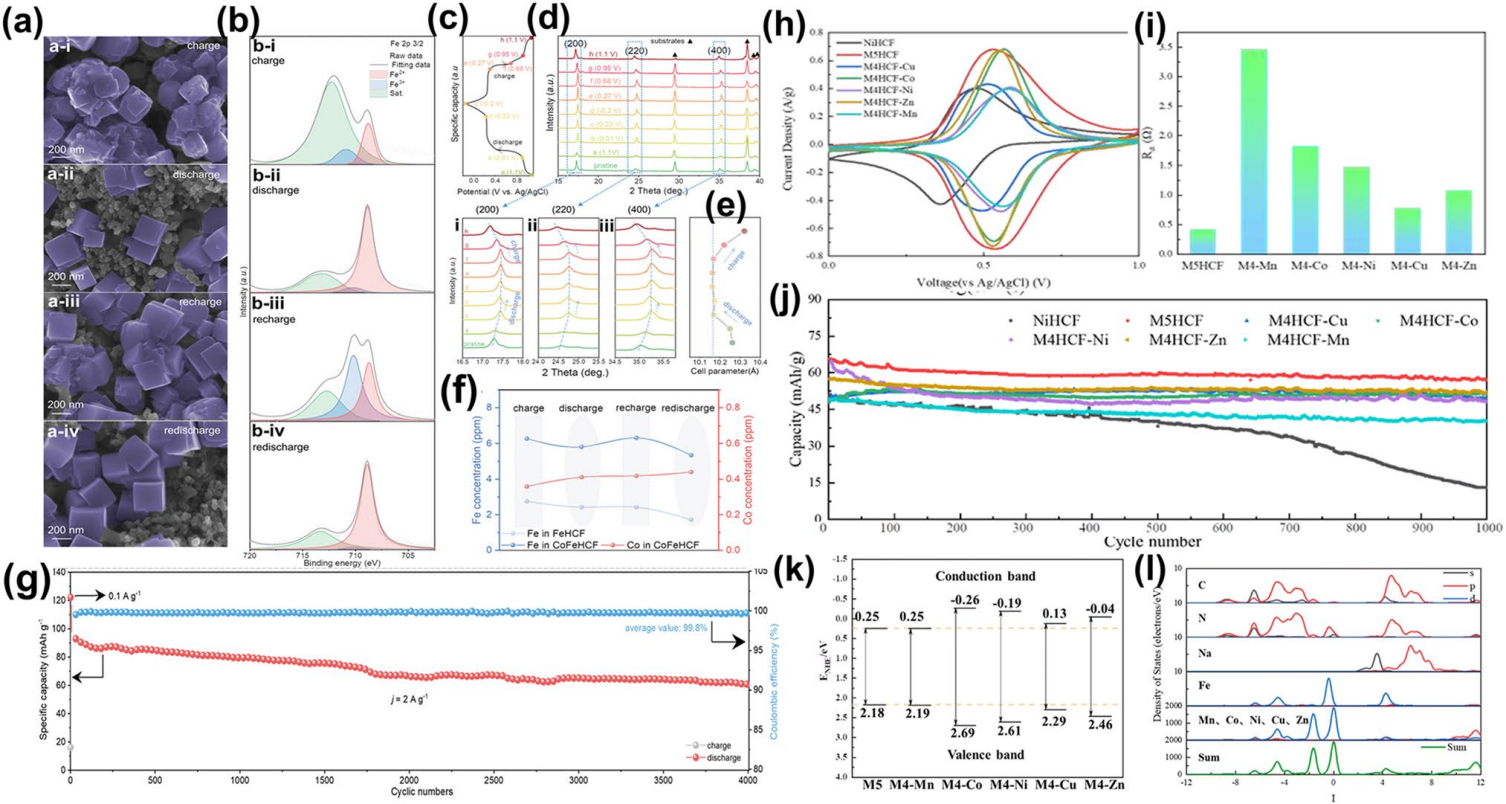
**a, b** SEM images and XPS spectra of CoFeHCF in different charge/discharge states. **c** Corresponding charge/discharge curves. **d** XRD pattern variations and **e** the corresponding cell parameters. **f** Variations in the concentration of Fe/Co in the electrolytes for CoFeHCF during cycles 21 and 22. **g** Long-term cyclic performance of CoFeHCF at 2 A g^−1^ [103]. Copyright 2022, Wiley–VCH. **h** CV curves of NiHCF, M5HCF and medium-entropy PBAs at 2 mV s^−1^. **i** Statistic results of *R*_*ct*_ values in DRT analysis. **j** Cycling performance at 1 C of NiHCF, M5HCF and medium-entropy PBAs cathode in PBAs||NTP@C full cells. **k** Schematic diagram of the band edges for M5HCF and medium-entropy doped PBAs. **l** DOS and PDOS plots of M5HCF [109]. Copyright 2023, The Royal Society of Chemistry

Chung et al. introduced V-doped V/Fe-PBAs materials, noting a substantial enhancement in electrochemical performance compared to Cu/Fe-PBAs and Ni/Fe-PBAs, ascribed to the structural robustness of V/Fe-PBAs electrodes and minimal lattice alterations during cycling [102]. However, ICP analyses revealed a progressive dissolution of V and Fe ions during cycling, initiating a capacity reduction at the cycle's outset. Conversely, Zhang et al. employed selective Co doping in minimal concentrations into CoFeHCF to counteract the PBAs framework's incremental degradation due to electrochemical cycling [103]. The ‘electrochemically driven dissolution–recrystallization’ self-healing mechanism endowed CoFeHCF with an increasingly organized structure, preserving an impeccable cubic morphology over numerous cycles. The morphological evolution of the CoFeHCF electrode through charge–discharge sequences under electric field influence is depicted in Fig. 7a. X-ray photoelectron spectroscopy (XPS) analyses indicated the attenuation of Fe^2+^ characteristic peaks in CoFeHCF in its deprotonated state, strengthening in the potassium form throughout the cycles (Fig. 7b). Furthermore, ex situ XRD patterns (Fig. 7c and d) documented the crystal structure's evolution during the electric field's influence, evidencing a solid solution mechanism devoid of phase transitions, accompanied by negligible lattice distortion and volume dynamics (Fig. 7e). This reversible cycle contributes significantly to the sustenance of structural integrity and electrochemical resilience. Experimental validations, allied with multiphysics simulations (MD) and density functional theory (DFT) analyses, postulate that Co incorporation within the PBA matrix could thwart Fe migration on the crystal facade, particularly in deteriorated areas, thereby recuperating crystallographic contours. The inherent ‘electrochemically driven dissolution–recrystallization’ underscores the pivotal role of the electric field in mediating reversible crystal growth. Co-integration optimally modulates the electrical reactivity of FeLS-C octahedra, fostering the self-healing attribute. Additionally, the enhanced conductivity and K-ion mobility attributable to Co doping amplify the cycling proficiency of CoFeHCF electrodes in dilute electrolytes, demonstrated over 4000 cycles (Fig. 7g). In a related study, Wang et al. incorporated Fe into CoHCF, synthesizing K_1.66_Fe_0.25_Co_0.75_[Fe(CN)_6_]·0.83H_2_O (KFCHCF) for APIBs [104]. This novel cell employing a KFCHCF cathode sustained 74.35% of its discharge capacity post 10,000 cycles at 1 A g^–1^. Furthermore, Reguera et al. mitigated structural perturbations by substituting MnHCF with high polarizability Co, modulating the electronic environment around Fe (LS) via cyanide connectors within the Mn-NC-Fe-CN-Co sequence [105]. This Co and Mn interaction in Co_0.55_Mn_0.45_HCF facilitated through the cyanide bridge fortified the redox constancy of the discharge voltage and overall electrochemical behavior, achieving 80% capacity preservation post 100 cycles.

In the domain of battery materials, the incorporation of a single TM often falls short of achieving desirable electrochemical attributes. Recent insights have underscored the potency of high-entropy compounds—comprising five or more elements—in augmenting the capacity and cycling robustness of electrode materials. These improvements are attributable to the entropic stability, lattice distortion, retarded ion diffusion, and cocktail effects inherent in these compounds [106, 107]. For instance, Xing et al. integrated Mn, Co, Ni, Cu, and Fe into the PBAs for aqueous series zinc-ion batteries. This incorporation of multiple transition metals at the active sites markedly curtailed PBA dissolution, thereby enhancing cycling stability [108]. Similarly, Huang et al. employed a high-entropy approach to craft high-entropy PBA (HEPBA; M5HCF) for use as a cathode material in ASIBs [109]. Evident in Fig. 7h, M5HCF exhibited an elevated redox potential within the 0–1 V range, coupled with a negligible redox peak potential discrepancy, underpinning its commendable reversibility. The absence of conspicuous H_2_ and O_2_ evolution underscored the reversibility of the Na^+^ insertion/extraction reaction. Remarkably, the cocktail effect induced the convergence of M5HCF's redox peaks into a single, broadened peak, with a redox peak potential difference of a mere 0.002 V, bolstering high reversibility and cycle durability. Electrochemical impedance spectroscopy (EIS) and distribution of relaxation times (DRT) analyses confirmed that M5HCF possessed minimal charge transfer and diffusion resistance, a characteristic pivotal for superior electrochemical behavior (Fig. 7i). DFT calculations revealed that high-entropy doping substantially constricted the band gap, simplifying electron excitation into the conduction band, and bolstering electron mobility. The reduction potential (*E*_c_) of the conduction band edge and the oxidation potential (*E*_v_) of the valence band edge is displayed in Fig. 7k. It is worth noting that the electron excitation and leaps are favored by the narrow band edge width of M5HCF. Furthermore, the total density of states (DOS) and partial density of state (PDOS) of M5HCF and medium-entropy PBAs are calculated in Fig. 7l. Those calculations confirm that doping with high entropy can enhance the electron density and conductivity of PBAs, also have a great influence on electrochemical performance at high rates.

TM doping or substitution methods used in elemental adjustments can regulate interatomic interactions, ultimately impacting the inherent stability of materials. In particular, the substitution of manganese with iron, nickel, or cobalt in Mn-based PBAs can minimize cathode degradation caused by disproportionation reactions and the Jahn–Teller distortion. Present advances in high-entropy doping help decrease the water affinity of PBAs and regulate their electronic spin states, thus boosting their electrochemical properties. Nonetheless, substituting Mn with inactive TM results in lost energy density and significantly higher costs of metal salts such as Ni and Co compared to Mn. More importantly, due to its promising adaptability, it is worthwhile to balance the relationship between energy density, cycle life, and manufacturing cost by utilizing TM doping or substitution, in various application environments.

### Minimize Defects

The enhancement of structural stability in electrode materials is often linked to high crystallinity, which is evident in various systems such as chalcogenides, conducting polymers, and organic superconductors [110–112]. To address the issue of TM ion dissolution and thereby bolster both structural and cyclic resilience, it is imperative to engineer PBA materials of superior crystallinity. Present synthesis methodologies for PBAs predominantly encompass the self-decomposition of an exclusive iron source and co-precipitation in water. The self-decomposition approach, albeit slower in reaction kinetics, permits facile control over particle morphology and size; however, it is restrictive, allowing the synthesis of only FeHCF. Conversely, the prevalent technique involves the aqueous co-precipitation of a TM solution with a cyanide anion solution. Despite its advantages—low cost, scalability, and versatility in obtaining PBAs of varied chemical compositions—this method is not without shortcomings. The minuscule solubility product constants of PBAs in water necessitate exceedingly rapid crystal nucleation and growth, consequently leading to substantial crystal defects and vacancies in Fe(CN)_6_, coordinated by lattice water. These inherent imperfections significantly undermine the stability of PBAs, facilitating the accelerated dissolution of TM ions. Given these dynamics, the strategic synthesis of ultra-low-defect PBAs becomes paramount in mitigating the challenges posed by TM ion dissolution. It is essential to refine existing methodologies or innovate new synthetic strategies to enhance the precision and control of crystal growth, thereby improving the performance metrics of PBA-based electrode materials.

**Fig. 8:**
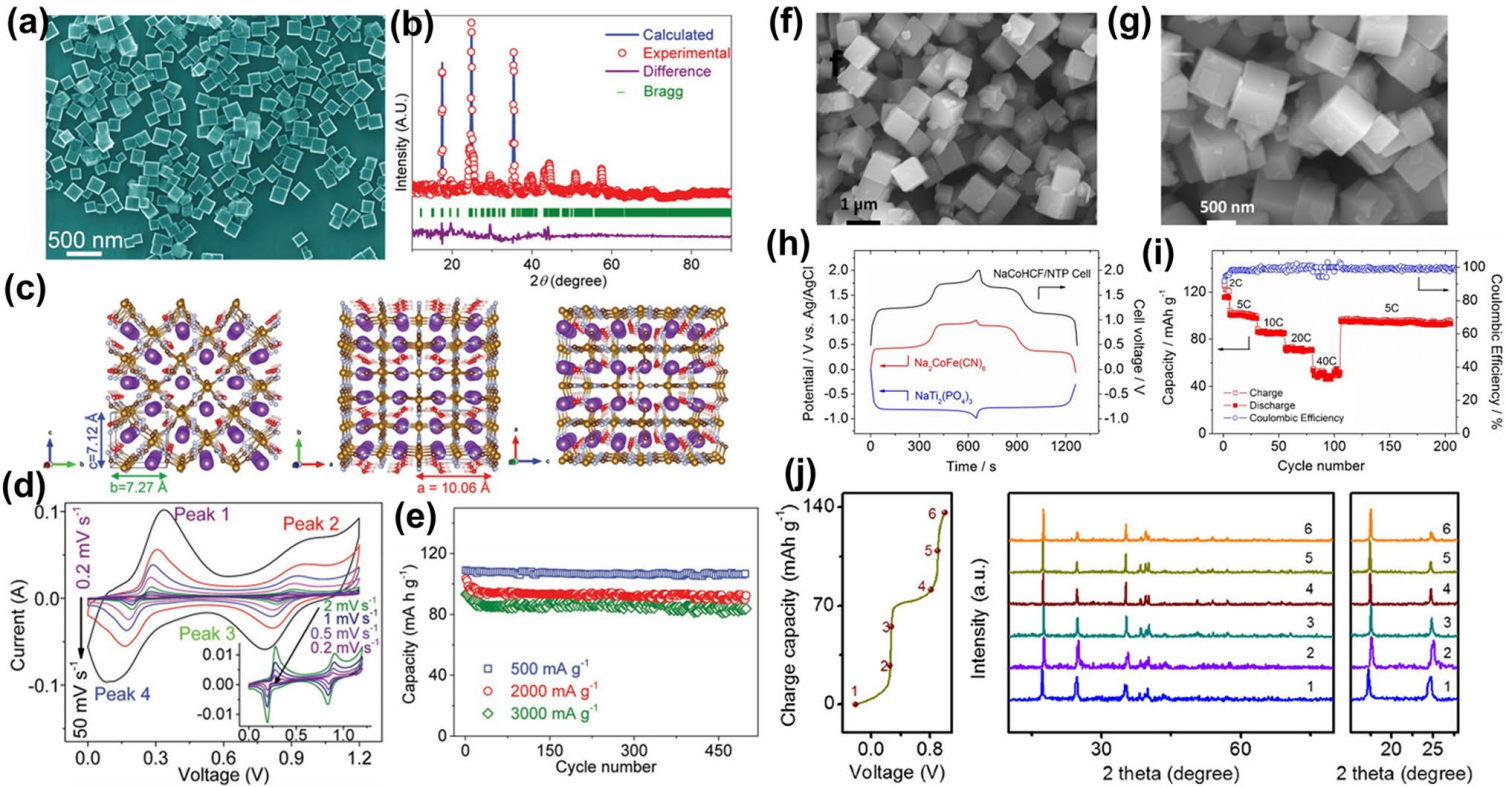
**a** SEM image of KFeHCF decomposition in ethylene glycol solution. **b** Rietveld refinement pattern of XRD for KFeHCF. **c** Refined crystal structure views along different axes. **d** CV images at different scanning rates. **e** Cycling performance of KFeHCF at different current densities [113]. Copyright 2017, Wiley–VCH. **f, g** SEM images of NaCoHCF. **h** Charge/discharge curves of the NaCoHCF||NaTi_2_(PO_4_)_3_ full cell at 5 C. **i** Rate and cycling performance [61]. Copyright 2015, Wiley–VCH. **j** Ex situ XRD patterns of KFe(1.0) electrode collected at various charged states [116]. Copyright 2018, Wiley–VCH

Wang et al. reported the synthesis of K_2_Fe^II^[Fe^II^(CN)_6_]·2H_2_O nanocubes, characterized by an open backbone structure, via a low-temperature solvothermal method employing ethylene glycol as the solvent [113]. This transition from water to ethylene glycol leveraged the solvent's reductive properties to prevent the oxidation of Fe^2+^. The resultant K_2_Fe^II^[Fe^II^(CN)_6_]·2H_2_O manifested as homogeneous nanocubes, each with an approximate side length of 200 nm (Fig. 8a). Subsequent Rietveld-refined X-ray diffraction analysis revealed the crystal structure identifying it as an orthorhombic symmetric formation within the Pmn21 space group (Fig. 8b). The refined structural model, displayed in Fig. 8c, demonstrated an octahedral framework, wherein two additional Fe atoms were alternately coordinated with carbon and nitrogen. Further electrochemical investigations, depicted in Fig. 8d, highlighted the material's expedited electrochemical kinetics and minimal polarization in an aqueous electrolyte, as evidenced by multi-sweep cyclic voltammetry. The endurance test underscored the compound's robust reversibility, maintaining performance over 500 cycles at varying current densities of 500, 2000, and 3000 mA g^–1^ (Fig. 8e).

Yang et al. delineated the fabrication of defect-free Na_2_CoFe(CN)_6_ nanocubes using a citrate chelator and explored their electrochemical characteristics in ASIBs [61]. In the co-precipitation phase, citrate ions selectively chelate Co^2+^, creating cobalt–citrate complexes, thereby moderating the rapid precipitation dynamics to facilitate the formation of uniform nanocubes. SEM images (Fig. 8f and g) confirmed that the synthesized NaCoHCF particles comprised well-defined, discrete nanocubes measuring 300–600 nm in size. The charge/discharge assay of the assembled NaCoHCF||NaTi_2_(PO_4_)_3_ full cell, illustrated in Fig. 8h, demonstrated superior rate capability and cyclic durability (Fig. 8i). Subsequent application of Na_2_CoFe(CN)_6_ within organic SIB frameworks retained commendable sodium storage efficacy [114]. In a separate study, Cai et al. scrutinized the crystallinity of PB cathodes in influencing the electrochemical attributes of ASIBs [115]. High-crystallinity PB, attributed to its immaculate lattice structure and distinct nanocubic morphology, exhibited elevated discharge potential and enhanced cycle life. Concurrently, Xue et al. crafted PBAs under varying crystallinity by manipulating the acidic milieu, assessing their impact on APIBs [116]. Notably, KFe(1.0) (K_1.93_Fe[Fe(CN)_6_]_0.97_·1.82H_2_O) with pronounced crystallinity, composed of 50-nm microcrystals, registered the pinnacle of reversible capacity and performance diversity. Ex situ XRD analyses discerned negligible crystallographic alterations during K^+^ intercalation, with only marginal lattice parameter fluctuations, corroborating the structural integrity of the high-crystallinity KFe(1.0) (Fig. 7j).

The fabrication of single-crystal cathodes, characterized by micrometer-sized individual particles devoid of grain boundaries, has been advocated to enhance stability and dependability. This strategy has recently garnered significant interest, underscoring the imperative for a synthesis technique tailored to PBAs. By minimizing grain boundaries, single-crystal particles curtail side reactions, deter TM ion dissolution, and mitigate voltage degradation. Hu et al. pioneered the creation of inverse opal single-crystal PBAs, exploiting the solubility properties of PBAs in volatile HCl environments and subsequent crystallization within a template [117]. These single-crystal constructs augment the contact surface, interfacing the structural matrix with the external conductive network or electrolyte, markedly abbreviating the ion diffusion path from the surface to the internal matrix. Notably, within the cohort of APIBs, these single-crystal PBAs manifest exemplary electrochemical prowess, sustaining nearly intact capacity retention approaching 100% post 1000 cyclic repetitions under a load of 20 A g^–1^.

Furthermore, it is worth noting that not all defects have adverse effects. A rational structural design can be made in PBAs by incorporating controllable defects or vacancy engineering, which enhancing structural stability, improving dissolution, and offering novel ideas in further explorison [37, 117, 118]. Mai et al. discovered that they could impede the movement of Fe–C bonds by intentionally introducing Fe^III^ vacancies in PBAs. In addition, it effectively mitigated the lattice distortion of Fe–C octahedra, thus leading to improvements in PBAs' reversibility and cyclic stability [119]. In a similar study, Yang et al. utilized strong chelation to introduce manganese vacancies, V_Mn_, on the surface of MnHCF [66]. For this V_Mn_, it can impede Mn-N bond movement, thus preventing Jahn–Teller distortion of the Mn-N_6_ octahedra, causing a reversible phase shift in the structure from monoclinic, cubic, and orthorhombic phases and improving electrochemical stability.

Particularly, the defects inherent in the structure of PBAs increase their susceptibility to solubilization, not only hindering ion storage and transport but also impairing crystallinity and structural robustness. Meanwhile, this can expedite the rapid dissolution of TM ions. With the use of low-cost aqueous co-precipitation synthesis, it is feasible to work out a promising approach of reducing harmful structural defects in designing rational structures incorporating low-cost chelating agents. However, further research is required on the precise conformational relationships of PBAs, given the complex influencing factors in the synthesis process.

### Composite Materials

**Fig. 9:**
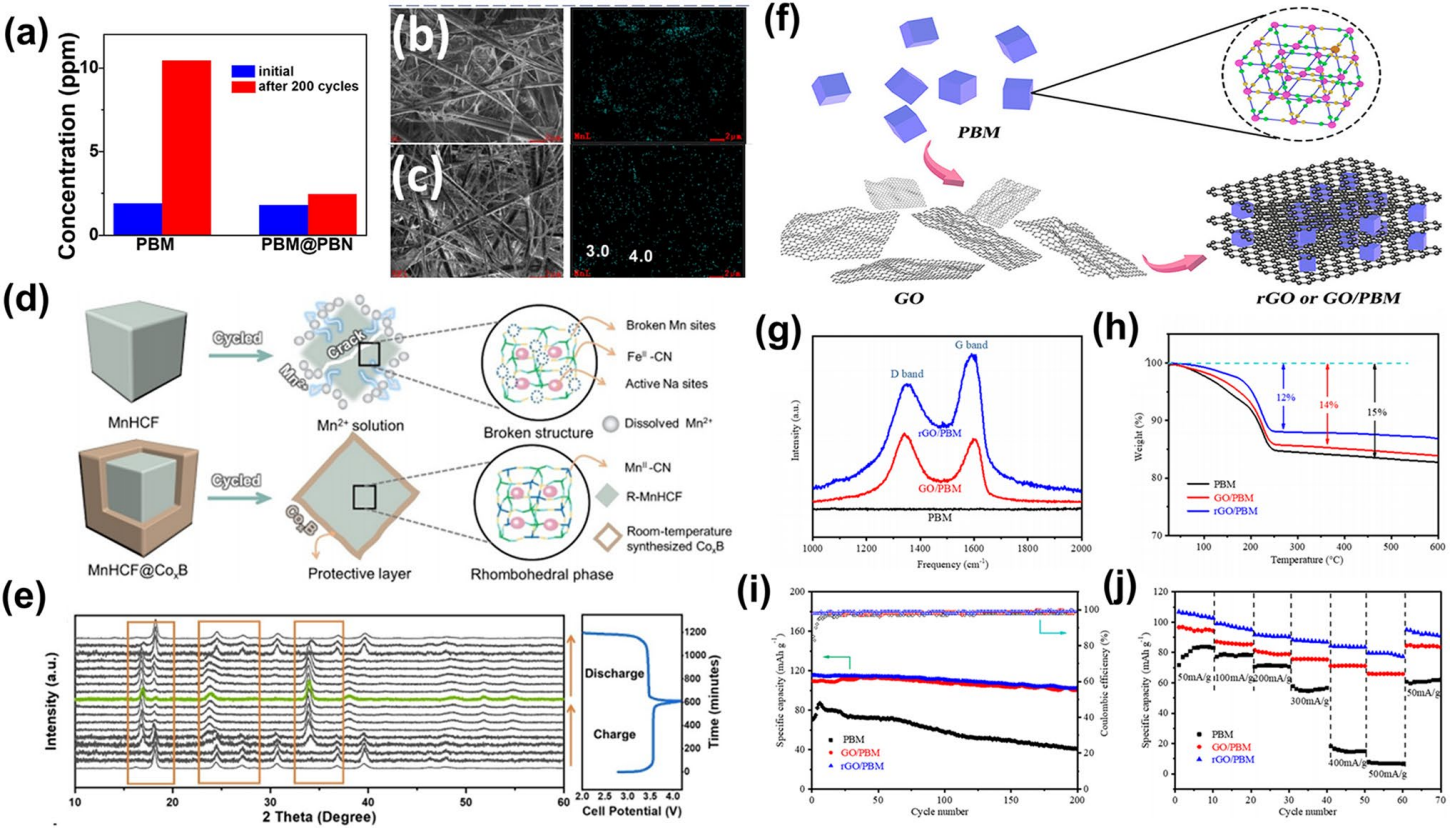
**a** Manganese dissolution in the electrolyte after long cycles. SEM and EDS mapping images of separators disassembled from **b** PBM and **c** PBM@PBN after 200 cycles, respectively [121]. Copyright 2021, Elsevier. **d** Schematic illustration of the proposed protective mechanism of CoχB to suppress the Mn dissolution and generation of microcracks of MnHCF. **e** Ex situ XRD patterns of the MnHCF-5%CoχB electrode during the first charge/discharge process [122]. Copyright 2023, Wiley–VCH. **f** Schematic illustration of PBM, GO/PBM, and rGO/PBM synthesis procedure. **g** Raman spectra. **h** TGA curves. **i** Cycling performance. **j** Rate capabilities [126]. Copyright 2021, American Chemical Society

The strategy of engineering composites grounded in PBAs presents a promising pathway to curtail TM ion migration efficaciously. Among various transition metal ions, the leaching of Mn ions into the electrolyte exhibits pronounced severity, necessitating a targeted approach for its mitigation. Liao et al. enhanced the electrochemical attributes of Na_2_Mn[Fe(CN)_6_] (PBM) through a meticulous solution–precipitation technique, encapsulating it with Na_3_(VOPO_4_)_2_F (NVOPF) [120]. This NVOPF encapsulation served to thwart the ancillary reactions between PBM and the electrolyte, concomitantly stalling the dissolution of Mn ions, thereby bolstering electrochemical efficacy. Subsequently, an advanced composite, PBM@PBN, was synthesized by overlaying PBM with Na_2_Ni[Fe(CN)_6_] (PBN) [121]. The PBN stratum not only impeded PBM's peripheral reactions with the electrolyte but also forestalled the seepage of Mn^2+^ ions therein, culminating in augmented multifaceted performance and extended battery longevity. As depicted in Fig. 9a, ICP analyses post-extended cycling sessions revealed a marked curtailment in electrolytic Mn. Initial dissolution rates of Mn from both PBM and PBM@PBN electrodes were akin. Nonetheless, after 200 cycles, PBM witnessed a quadrupled Mn dissolution rate relative to PBM@PBN, underscoring the inhibitory role of the PBN coating. Furthermore, energy-dispersive X-ray spectroscopy (EDS) mapping affirmed a higher residual Mn content on the PBM-separated membrane compared to its PBM@PBN counterpart (Fig. 9b and c). Hu et al. unveiled a simplistic aqueous phase coating technique, devising a comprehensive CoχB layer upon the MnHCF surface [122]. Owing to the all-encompassing nature and buffer capacity of the nanoscale CoχB layer, the tailored MnHCF hindered Mn ion dissolution, minimized intraparticle fissuring, and, thus, demonstrated endurance through thousands of cycles. Figure 9d vividly captures the CoχB's ameliorative impact on MnHCF's structural frailties and Mn ion dissolution throughout the cycling process. Ex situ XRD analyses revealed the MnHCF-5% CoχB electrode undergoing reversible phase modulations from rhombohedral to tetrahedral configurations amid Na^+^ intercalation/de-intercalation (Fig. 9e), validating the CoχB coating's efficiency in negating cubic particle deformation.

The development of composites based on PBAs presents a strategy for enhancing electrochemical attributes in ASIBs. For instance, Feng et al. enhanced the specific capacity and rate performance of Na_1.88_Fe[Fe(CN)_6_]·0.7H_2_O by integrating Ketjenblack through a ball milling process [123]. Similarly, Zarbin et al. synthesized carbon nanotube composite PBAs, where the formation of PB on individual CNTs facilitated slower Fe diffusion, thereby improving reversibility and stability in APIBSs [124]. This structural modification also conferred enhanced electronic conductivity, ameliorating charge transport limitations within the PBs. In another study, Coronado et al. developed PB/MoS_2_ nanocomposites, with MoS_2_ promoting uniform PB nucleation, which expanded the material's specific surface area [125]. This increased optimized PB-matrix contact, enhancing electron transport and structural resilience, reflected in superior cathodic properties for ASIBs and APIBs. Performance metrics indicated a discharge capacity of 177 mAh g^–1^ and a specific capacitance of 354 F g^–1^ at 1 A g^–1^ for ASIBs, with APIBs demonstrating improved values of 215 mAh g^–1^ and 489 F g^–1^ under identical conditions. Addressing PBM's electrical conductivity deficits, Li et al. incorporated GO and rGO to form GO/PBM and rGO/PBM composites, as depicted in Fig. 9f [126]. Spectroscopic analysis revealed an elevated G-band intensity for rGO/PBM relative to GO/PBM, suggesting augmented *sp*^2^-hybridized carbon concentration post-reduction, thereby bolstering rGO/PBM conductivity (Fig. 9g). Concurrently, diminished water content in both composites implied defect diminution (Fig. 9h). This structural refinement facilitated commendable cycling stability, retaining approximately 91% and 89% capacity over 200 cycles for GO/PBM and rGO/PBM, respectively (Fig. 9i). Notably, rGO inclusion substantially enhanced the active material's electrical conductivity, expediting Na^+^ intercalation/deintercalation and markedly boosting rate performance (Fig. 9j).

Combining the conductive material and PBA can create a protective layer on the surface of the electrode material, reducing the impact of reactive water on the PBA and minimizing lattice damage during charging and discharging cycles. This method addresses the issue of inadequate intrinsic conductivity in PBA, prevents material dissolution caused by structural collapse, and enhances the overall stability of the composite electrode. Since PBAs' poor intrinsic thermal stability decomposes at 350 °C, the limited selection of composite strategies is largely available. Thus, further research can be focused on identifying stable candidate materials in both electrode material and electrolyte to expand the options for stable composite formation with PBAs at lower temperatures.

## Conclusions and Outlook

In EESs, ASIBs and APIBs are gaining prominence owing to their cost-effectiveness, high-energy conversion efficiency, eco-friendliness, and enhanced safety features. PBAs are viewed as potential candidates for large-scale synthesis, attributed to their three-dimensional framework structures and economic viability. Nonetheless, practical applications of these materials are substantially hindered by the capacity decay and cycling stability degradation, consequences of TM ion dissolution during electrochemical cycling in aqueous environments. This review meticulously dissects the architecture of PBAs and the dissolution mechanisms of TM ions. Emphasis has been placed on various strategies to mitigate TM ion dissolution, encompassing electrolyte engineering, TM doping/substitution, minimize defects, and composite materials. Among them, electrolyte engineering is the most effective strategy to protect the cathode material from TM ion dissolution by directly reducing the active water in the external environment. Additionally, selecting suitable electrolytes and developing new ones are expected to achieve high-energy density while maintaining exceptional cycling performance. Furthermore, in order to mitigate irreversible phase transitions, it is suggested to optimize the chemical composition and morphological structure of PBAs through a TM doping/substitution strategy that effectively fine-tunes the lattice parameters and redox properties. Since the synthesized PBAs typically exhibit a range of defects that impede the structural integrity and compromise the electrochemical performance, minimizing defects is required to enhance the electrochemical performance of PBA electrodes. Furthermore, using composites with other conductive materials in PBA cathodes is common to minimize surface defects and enhance structural stability while improving conductivity. These strategies substantially reduce the rate of TM ion dissolution in PBAs, which improve electrochemical performance in both ASIBs and APIBs. Despite noteworthy advancements addressing TM ion dissolution within PBAs, more comprehensive studies are imperative to meet the rigorous demands of practical high-safety applications.

### Optimization of Electrolyte

Electrolyte design aims to reduce free water and control the dissolution equilibrium, requiring an analysis of parameters such as salt anions’ hydrophobicity, salt concentration, and salt solubility in aqueous solutions. For PBAs, they encounter restricted voltage windows and significant problems with TM ion dissolution during cycling in low salt concentration conventional electrolytes. Therefore, the importance of developing electrolytes that broaden voltage windows and hinder TM ion dissolution cannot be overstated. High salt concentrations are the most effective and efficient strategy for high-energy, stable aqueous batteries, though the cost may hinder larger-scale use. Additionally, non-flammable electrolytes with high WiSE concentrations can expand the electrochemical stability window and curb material dissolution. The high viscosity and slow interfacial processes impede electrode kinetics and ion transport, hindering high rate performance. Nonetheless, energy storage devices prioritize cycle stability and energy density over other metrics. Specifically, in situ surface modification and repair of PBA materials can be accomplished by introducing a cation trapping agent into the electrolyte to inhibit TM ion dissolution. Synergizing the WiSE technique with an in situ surface modification strategy can potentially optimize energy density and cycle stability, making it a promising avenue of research. Additionally, developing new ‘dilute’ high-concentration electrolyte systems could enhance competitiveness by reducing costs and improving both rate performance and cycle stability.

### Optimization of Materials

In general, defects in the synthesis process can lead to structural breakdown during subsequent cycles for PBA cathodes. Therefore, it is necessary to improve synthesis methods aiming to minimize these harmful defects. In addition, aqueous co-precipitation synthesis and appropriate chelating agents are crucial for low cost, large-scale synthesis. Moreover, the electrochemical framework can be designed and adjusted through doping/substitution strategies using TM. Particularly, MnHCF is advantageous due to its low cost and high-energy density. However, the dissolution of Mn has received great attention, especially the dissolution partially replacing Mn with inactive TM, which can be reduced. Structural distortion from the Jahn–Teller effect can also be reduced. In addition, it is of significant interest on the incorporation of trace Co doping for self-repair and the emergence of high-entropy PBAs. Furthermore, combining PBAs with highly conductive materials boosts conductivity, prevents material dissolution from structural collapse, and enhances composite electrode stability. The overall rate performance and cycling stability can be significantly improved by synergizing these strategies. Notably, there is a lack of comprehensive research on anode material modification strategies, specifically on TM deposition on SEI. This topic desperately requires the attention of professionals and academics.

### Advanced Characterization and Theoretical Calculations

The integration of advanced in situ characterization methodologies with theoretical computations serves as a pivotal auxiliary instrument, deepening the comprehension of electrochemical processes in PBAs for ASIBs and APIBs. Delving into the dissolution phenomena of TM ions mandates the utilization of refined in situ characterization techniques, including in situ XRD, TEM, XPS, Raman, and STEM, fostering an intricate understanding of the underpinning principles of TM ion dissolution. In parallel, molecular dynamics simulations prove instrumental in dissecting interfacial interactions between electrodes and electrolytes, thereby elucidating critical parameters within the TM ion dissolution trajectory. Additionally, first-principles calculations, particularly those concerning the density of states and ion diffusion barriers within PBA electrodes, are indispensable in uncovering inherent reaction pathways and crystallographic transformations during charge–discharge cycles. For the application of these advanced characterizations and computational tools, there is a deeper understanding of the TM dissolution mechanism that helps to overcome the electrochemical performance degradation caused by TM dissolution–migration–deposition.

### Limit Balance Control

In the strategic enhancement of battery design and management, cognizance of the aging mechanism and subsequent implications of battery degradation is paramount. Battery cycle life is subject to an array of influences. Notably, the cathode undergoes recurrent alterations in its material constitution throughout the charge/discharge cycles, culminating over time in structural disintegration, TM ion dissolution, and consequent capacity attrition. The attrition of electrolyte volume markedly impinges on the comprehensive battery lifespan, a scenario pronounced particularly in button and soft-pack batteries. Marginal electrolyte diminution marginally impacts battery functionality, whereas substantial electrolyte depletion precipitates an abrupt capacity regression. Furthermore, issues stemming from alkali metal deposition and solid electrolyte interphase formation contribute to expedited capacity deterioration, engendering safety concerns. Addressing battery degradation necessitates expanded research, with a substantial body of inquiry concentrating on the capacity degradation attributable to individual determinants. Unraveling the inherent correlation among the rates of cathode capacity decay, capacity decay, and electrolyte utilization is crucial for achieving equilibrium control at the battery's operational limit.

## References

[1] Z. Yang, J. Zhang, M.C.W. Kintner-Meyer, X. Lu, D. Choi et al., Electrochemical energy storage for green grid. Chem. Rev. **111**, 3577–3613 (2011). 10.1021/cr100290v 21375330 10.1021/cr100290v

[2] D. Larcher, J.-M. Tarascon, Towards greener and more sustainable batteries for electrical energy storage. Nat. Chem. **7**, 19–29 (2015). 10.1038/nchem.2085 25515886 10.1038/nchem.2085

[3] X.-T. Wang, Z.-Y. Gu, E.H. Ang, X.-X. Zhao, X.-L. Wu et al., Prospects for managing end-of-life lithium-ion batteries: present and future. Interdiscip. Mater. **1**, 417–433 (2022). 10.1002/idm2.12041

[4] D. Chao, W. Zhou, F. Xie, C. Ye, H. Li et al., Roadmap for advanced aqueous batteries: from design of materials to applications. Sci. Adv. **6**, eaba4098 (2020). 10.1126/sciadv.aba4098 32494749 10.1126/sciadv.aba4098 PMC7244306

[5] N. Nitta, F. Wu, J.T. Lee, G. Yushin, Li-ion battery materials: present and future. Mater. Today **18**, 252–264 (2015). 10.1016/j.mattod.2014.10.040

[6] K. Liu, Y. Liu, D. Lin, A. Pei, Y. Cui, Materials for lithium-ion battery safety. Sci. Adv. **4**, eaas9820 (2018). 10.1126/sciadv.aas9820 29942858 10.1126/sciadv.aas9820 PMC6014713

[7] H.-J. Liang, Z.-Y. Gu, X.-X. Zhao, J.-Z. Guo, J.-L. Yang et al., Advanced flame-retardant electrolyte for highly stabilized K-ion storage in graphite anode. Sci. Bull. **67**, 1581–1588 (2022). 10.1016/j.scib.2022.07.002 10.1016/j.scib.2022.07.002 36546286

[8] R.Y. Wang, C.D. Wessells, R.A. Huggins, Y. Cui, Highly reversible open framework nanoscale electrodes for divalent ion batteries. Nano Lett. **13**, 5748–5752 (2013). 10.1021/nl403669a 24147617 10.1021/nl403669a

[9] K. Wang, H. Li, Z. Xu, H. Wang, M. Ge et al., Emerging photo-integrated rechargeable aqueous zinc-ion batteries and capacitors toward direct solar energy conversion and storage. Carbon Neutr. **2**, 37–53 (2023). 10.1002/cnl2.41

[10] M. Zhu, H. Wang, W. Lin, D. Chan, H. Li et al., Amphipathic molecules endowing highly structure robust and fast kinetic vanadium-based cathode for high-performance zinc-ion batteries. Small Struct. **3**, 2200016 (2022). 10.1002/sstr.202200016

[11] H. Wang, H. Li, Y. Tang, Z. Xu, K. Wang et al., Stabilizing Zn anode interface by simultaneously manipulating the thermodynamics of Zn nucleation and overpotential of hydrogen evolution. Adv. Funct. Mater. **32**, 2270271 (2022). 10.1002/adfm.202270271

[12] D. Xie, Y. Sang, D.-H. Wang, W.-Y. Diao, F.-Y. Tao et al., ZnF_2_-Riched inorganic/organic hybrid Sei: in situ-chemical construction and performance-improving mechanism for aqueous zinc-ion batteries. Angew. Chem. Int. Ed. **62**, e202216934 (2023). 10.1002/anie.202216934 10.1002/anie.202216934 36478517

[13] J. Jiang, J. Liu, Iron anode-based aqueous electrochemical energy storage devices: recent advances and future perspectives. Interdiscip. Mater. **1**, 116–139 (2022). 10.1002/idm2.12007

[14] J.-L. Yang, J.-M. Cao, X.-X. Zhao, K.-Y. Zhang, S.-H. Zheng et al., Advanced aqueous proton batteries: working mechanism, key materials, challenges and prospects. EnergyChem **4**, 100092 (2022). 10.1016/j.enchem.2022.100092

[15] G. Yang, Y. Zhu, Q. Zhao, Z. Hao, Y. Lu et al., Advanced organic electrode materials for aqueous rechargeable batteries. Sci. China Chem. (2023). 10.1007/s11426-023-1654-5

[16] C. Deng, Y. Li, J. Huang, Building smarter aqueous batteries. Small. Methods (2023). 10.1002/smtd.202300832 10.1002/smtd.202300832 37670546

[17] W. Li, J.R. Dahn, D.S. Wainwright, Rechargeable lithium batteries with aqueous electrolytes. Science **264**, 1115–1118 (1994). 10.1126/science.264.5162.1115 17744893 10.1126/science.264.5162.1115

[18] K. Kubota, M. Dahbi, T. Hosaka, S. Kumakura, S. Komaba, Towards K-ion and Na-ion batteries as “beyond Li-ion.” Chem. Rec. **18**, 459–479 (2018). 10.1002/tcr.201700057 29442429 10.1002/tcr.201700057

[19] T. Hosaka, K. Kubota, A.S. Hameed, S. Komaba, Research development on K-ion batteries. Chem. Rev. **120**, 6358–6466 (2020). 10.1021/acs.chemrev.9b00463 31939297 10.1021/acs.chemrev.9b00463

[20] F. Wan, Z. Niu, Design strategies for vanadium-based aqueous zinc-ion batteries. Angew. Chem. Int. Ed. **58**, 16358–16367 (2019). 10.1002/anie.201903941 10.1002/anie.201903941 31050086

[21] Y. Lu, X. Wu, Z. Li, H. Jiang, L. Liu et al., Na+/K+-codoped amorphous manganese oxide with enhanced performance for aqueous sodium-ion battery. J. Alloys Compd. **937**, 168344 (2023). 10.1016/j.jallcom.2022.168344

[22] C. Zhao, Q. Wang, Z. Yao, J. Wang, B. Sánchez-Lengeling et al., Rational design of layered oxide materials for sodium-ion batteries. Science **370**, 708–711 (2020). 10.1126/science.aay9972 33154140 10.1126/science.aay9972

[23] X. Zhang, X. Yang, G. Sun, S. Yao, Y. Xie et al., Hydration enables air-stable and high-performance layered cathode materials for both organic and aqueous potassium-ion batteries. Adv. Funct. Mater. **32**, 2204318 (2022). 10.1002/adfm.202204318

[24] L. Sharma, A. Manthiram, Polyanionic insertion hosts for aqueous rechargeable batteries. J. Mater. Chem. A **10**, 6376–6396 (2022). 10.1039/D1TA11080B

[25] H. Zhang, X. Tan, H. Li, S. Passerini, W. Huang, Assessment and progress of polyanionic cathodes in aqueous sodium batteries. Energy Environ. Sci. **14**, 5788–5800 (2021). 10.1039/D1EE01392K

[26] K.-Y. Zhang, Z.-Y. Gu, E.H. Ang, J.-Z. Guo, X.-T. Wang et al., Advanced polyanionic electrode materials for potassium-ion batteries: progresses, challenges and application prospects. Mater. Today **54**, 189–201 (2022). 10.1016/j.mattod.2022.02.013

[27] K. Holguin, M. Mohammadiroudbari, K. Qin, C. Luo, Organic electrode materials for non-aqueous, aqueous, and all-solid-state Na-ion batteries. J. Mater. Chem. A **9**, 19083–19115 (2021). 10.1039/D1TA00528F

[28] S. Zhang, C. Zhao, K. Zhu, J. Zhao, Y. Gao et al., An environment-friendly high-performance aqueous Mg-Na hybrid-ion battery using an organic polymer anode. Energy Environ. Mater. **6**, 12388 (2023). 10.1002/eem2.12388

[29] R. Wang, M. Shi, L. Li, Y. Zhao, L. Zhao et al., *In-situ* investigation and application of cyano-substituted organic electrode for rechargeable aqueous Na-ion batteries. Chem. Eng. J. **451**, 138652 (2023). 10.1016/j.cej.2022.138652

[30] K. Nakamoto, R. Sakamoto, Y. Sawada, M. Ito, S. Okada, Over 2 V aqueous sodium-ion battery with Prussian blue-type electrodes. Small Meth. **3**, 1800220 (2019). 10.1002/smtd.201800220

[31] L. Jiang, Y. Lu, C. Zhao, L. Liu, J. Zhang et al., Building aqueous K-ion batteries for energy storage. Nat. Energy **4**, 495–503 (2019). 10.1038/s41560-019-0388-0

[32] J. Ge, L. Fan, A.M. Rao, J. Zhou, B. Lu, Surface-substituted Prussian blue analogue cathode for sustainable potassium-ion batteries. Nat. Sustain. **5**, 225–234 (2022). 10.1038/s41893-021-00810-7

[33] C. Xu, Z. Yang, X. Zhang, M. Xia, H. Yan et al., Prussian blue analogues in aqueous batteries and desalination batteries. Nano-Micro Lett. **13**, 166 (2021). 10.1007/s40820-021-00700-9 10.1007/s40820-021-00700-9 PMC8342658 34351516

[34] A. Simonov, T. De Baerdemaeker, H.L.B. Boström, M.L. Ríos Gómez, H.J. Gray et al., Hidden diversity of vacancy networks in Prussian blue analogues. Nature **578**, 256–260 (2020). 10.1038/s41586-020-1980-y 32051599 10.1038/s41586-020-1980-y PMC7025896

[35] J. Peng, W. Zhang, Q. Liu, J. Wang, S. Chou et al., Prussian blue analogues for sodium-ion batteries: past, present, and future. Adv. Mater. **34**, e2108384 (2022). 10.1002/adma.202108384 34918850 10.1002/adma.202108384

[36] P.N. Le Pham, R. Wernert, M. Cahu, M.T. Sougrati, G. Aquilanti et al., Prussian blue analogues for potassium-ion batteries: insights into the electrochemical mechanisms. J. Mater. Chem. A **11**, 3091–3104 (2023). 10.1039/d2ta08439b

[37] Z. Wang, W. Zhuo, J. Li, L. Ma, S. Tan et al., Regulation of ferric iron vacancy for Prussian blue analogue cathode to realize high-performance potassium ion storage. Nano Energy **98**, 107243 (2022). 10.1016/j.nanoen.2022.107243

[38] C. Ding, Z. Chen, C. Cao, Y. Liu, Y. Gao, Advances in Mn-based electrode materials for aqueous sodium-ion batteries. Nano-Micro Lett. **15**, 192 (2023). 10.1007/s40820-023-01162-x 10.1007/s40820-023-01162-x PMC10412524 37555908

[39] W. Zhuo, J. Li, X. Li, L. Ma, G. Yan et al., Improving rechargeability of Prussian blue cathode by graphene as conductive agent for sodium ion batteries. Surf. Interfaces **23**, 100911 (2021). 10.1016/j.surfin.2020.100911

[40] W. Shu, C. Han, X. Wang, Prussian blue analogues cathodes for nonaqueous potassium-ion batteries: past, present, and future. Adv. Funct. Mater. (2023). 10.1002/adfm.202309636

[41] V.D. Neff, Electrochemical oxidation and reduction of thin films of Prussian blue. J. Electrochem. Soc. **125**, 886–887 (1978). 10.1149/1.2131575

[42] D. Ellis, M. Eckhoff, V.D. Neff, Electrochromism in the mixed-valence hexacyanides. 1. Voltammetric and spectral studies of the oxidation and reduction of thin films of Prussian blue. J. Phys. Chem. **85**, 1225–1231 (1981). 10.1021/j150609a026

[43] K. Itaya, T. Ataka, S. Toshima, Spectroelectrochemistry and electrochemical preparation method of Prussian blue modified electrodes. J. Am. Chem. Soc. **104**(18), 4767–4772 (1982). 10.1021/ja00382a006

[44] A.A. Karyakin, Prussian blue and its analogues: electrochemistry and analytical applications. Electroanalysis **13**, 813–819 (2001). 10.1002/1521-4109

[45] M. Pasta, C.D. Wessells, R.A. Huggins, Y. Cui, A high-rate and long cycle life aqueous electrolyte battery for grid-scale energy storage. Nat. Commun. **3**, 1149 (2012). 10.1038/ncomms2139 23093186 10.1038/ncomms2139

[46] C.D. Wessells, R.A. Huggins, Y. Cui, Copper hexacyanoferrate battery electrodes with long cycle life and high power. Nat. Commun. **2**, 550 (2011). 10.1038/ncomms1563 22109524 10.1038/ncomms1563

[47] G. Fang, Q. Wang, J. Zhou, Y. Lei, Z. Chen et al., Metal organic framework-templated synthesis of bimetallic selenides with rich phase boundaries for sodium-ion storage and oxygen evolution reaction. ACS Nano **13**, 5635–5645 (2019). 10.1021/acsnano.9b00816 31022345 10.1021/acsnano.9b00816

[48] J. Li, P. Ruan, X. Chen, S. Lei, B. Lu et al., Aqueous batteries for human body electronic devices. ACS Energy Lett. **8**, 2904–2918 (2023). 10.1021/acsenergylett.3c00678

[49] S. Qiu, Y. Xu, X. Wu, X. Ji, Prussian blue analogues as electrodes for aqueous monovalent ion batteries. Electrochem. Energy Rev. **5**, 242–262 (2022). 10.1007/s41918-020-00088-x

[50] A. Zhou, W. Cheng, W. Wang, Q. Zhao, J. Xie et al., Hexacyanoferrate-type Prussian blue analogs: principles and advances toward high-performance sodium and potassium ion batteries. Adv. Energy Mater. **11**, 2000943 (2021). 10.1002/aenm.202000943

[51] D. Kim, T. Hwang, J.-M. Lim, M.-S. Park, M. Cho et al., Hexacyanometallates for sodium-ion batteries: insights into higher redox potentials using d electronic spin configurations. Phys. Chem. Chem. Phys. **19**, 10443–10452 (2017). 10.1039/c7cp00378a 28379270 10.1039/c7cp00378a

[52] A. Kumar, S.M. Yusuf, L. Keller, Structural and magnetic properties of Fe[Fe(CN)6]·4H_2_O. Phys. Rev. B **71**, 054414 (2005). 10.1103/PhysRevB.71.054414

[53] N. Shimamoto, S.-I. Ohkoshi, O. Sato, K. Hashimoto, Control of charge-transfer-induced spin transition temperature on cobalt-iron Prussian blue analogues. Inorg. Chem. **41**, 678–684 (2002). 10.1021/ic010915u 11849066 10.1021/ic010915u

[54] A. Paolella, C. Faure, V. Timoshevskii, S. Marras, G. Bertoni et al., A review on hexacyanoferrate-based materials for energy storage and smart windows: challenges and perspectives. J. Mater. Chem. A **5**, 18919–18932 (2017). 10.1039/C7TA05121B

[55] H.J. Buser, D. Schwarzenbach, W. Petter, A. Ludi, The crystal structure of Prussian Blue: Fe4[Fe(CN)6]_3_.xH_2_O. Inorg. Chem. **16**, 2704–2710 (1977). 10.1021/ic50177a008

[56] J. Sun, H. Ye, J.A.S. Oh, A. Plewa, Y. Sun et al., Elevating the discharge plateau of Prussian blue analogs through low-spin fe redox induced intercalation pseudocapacitance. Energy Storage Mater. **43**, 182–189 (2021). 10.1016/j.ensm.2021.09.004

[57] M. Jiang, Z. Hou, L. Ren, Y. Zhang, J.-G. Wang, Prussian blue and its analogues for aqueous energy storage: from fundamentals to advanced devices. Energy Storage Mater. **50**, 618–640 (2022). 10.1016/j.ensm.2022.06.006

[58] L.-P. Wang, P.-F. Wang, T.-S. Wang, Y.-X. Yin, Y.-G. Guo et al., Prussian blue nanocubes as cathode materials for aqueous Na-Zn hybrid batteries. J. Power. Sources **355**, 18–22 (2017). 10.1016/j.jpowsour.2017.04.049

[59] Z. Wang, Y. Huang, D. Chu, C. Li, Y. Zhang et al., Continuous conductive networks built by Prussian blue cubes and mesoporous carbon lead to enhanced sodium-ion storage performances. ACS Appl. Mater. Interfaces **13**, 38202–38212 (2021). 10.1021/acsami.1c06634 34342988 10.1021/acsami.1c06634

[60] T. Shao, C. Li, C. Liu, W. Deng, W. Wang et al., Electrolyte regulation enhances the stability of Prussian blue analogues in aqueous Na-ion storage. J. Mater. Chem. A **7**, 1749–1755 (2019). 10.1039/C8TA10860A

[61] X. Wu, M. Sun, S. Guo, J. Qian, Y. Liu et al., Vacancy-free Prussian blue nanocrystals with high capacity and superior cyclability for aqueous sodium-ion batteries. ChemNanoMat **1**, 188–193 (2015). 10.1002/cnma.201500021

[62] L. Wang, J. Song, R. Qiao, L.A. Wray, M.A. Hossain et al., Rhombohedral Prussian white as cathode for rechargeable sodium-ion batteries. J. Am. Chem. Soc. **137**, 2548–2554 (2015). 10.1021/ja510347s 25615887 10.1021/ja510347s

[63] M. Qin, W. Ren, J. Meng, X. Wang, X. Yao et al., Realizing superior Prussian blue positive electrode for potassium storage via ultrathin nanosheet assembly. ACS Sustain. Chem. Eng. **7**, 11564–11570 (2019). 10.1021/acssuschemeng.9b01454

[64] A. Zhou, Z. Xu, H. Gao, L. Xue, J. Li et al., Size-, water-, and defect-regulated potassium manganese hexacyanoferrate with superior cycling stability and rate capability for low-cost sodium-ion batteries. Small **15**, e1902420 (2019). 10.1002/smll.201902420 31469502 10.1002/smll.201902420

[65] L. Deng, J. Qu, X. Niu, J. Liu, J. Zhang et al., Defect-free potassium manganese hexacyanoferrate cathode material for high-performance potassium-ion batteries. Nat. Commun. **12**, 2167 (2021). 10.1038/s41467-021-22499-0 33846311 10.1038/s41467-021-22499-0 PMC8041879

[66] Y. Shang, X. Li, J. Song, S. Huang, Z. Yang et al., Unconventional Mn vacancies in Mn-Fe Prussian blue analogs: suppressing jahn-teller distortion for ultrastable sodium storage. Chem **6**, 1804–1818 (2020). 10.1016/j.chempr.2020.05.004

[67] F. Gebert, D.L. Cortie, J.C. Bouwer, W. Wang, Z. Yan et al., Epitaxial nickel ferrocyanide stabilizes jahn-teller distortions of manganese ferrocyanide for sodium-ion batteries. Angew. Chem. Int. Ed. **60**, 18519–18526 (2021). 10.1002/anie.202106240 10.1002/anie.202106240 34096153

[68] L. Shen, Y. Jiang, Y. Liu, J. Ma, T. Sun et al., High-stability monoclinic nickel hexacyanoferrate cathode materials for ultrafast aqueous sodium ion battery. Chem. Eng. J. **388**, 124228 (2020). 10.1016/j.cej.2020.124228

[69] W. Ren, X. Chen, C. Zhao, Ultrafast aqueous potassium-ion batteries cathode for stable intermittent grid-scale energy storage. Adv. Energy Mater. **8**, 1801413 (2018). 10.1002/aenm.201801413

[70] S.-B. Son, Z. Zhang, J. Gim, C.S. Johnson, Y. Tsai et al., Transition metal dissolution in lithium-ion cells: a piece of the puzzle. J. Phys. Chem. C **127**, 1767–1775 (2023). 10.1021/acs.jpcc.2c08234

[71] Y. Zhang, A. Hu, D. Xia, S. Hwang, S. Sainio et al., Operando characterization and regulation of metal dissolution and redeposition dynamics near battery electrode surface. Nat. Nanotechnol. **18**, 790–797 (2023). 10.1038/s41565-023-01367-6 37081082 10.1038/s41565-023-01367-6

[72] D.H. Jang, Y.J. Shin, S.M. Oh, Dissolution of spinel oxides and capacity losses in 4 V Li / Li x Mn2 O 4 cells. J. Electrochem. Soc. **143**, 2204–2211 (1996). 10.1149/1.1836981

[73] X. Gao, Y.H. Ikuhara, C.A.J. Fisher, R. Huang, A. Kuwabara et al., Oxygen loss and surface degradation during electrochemical cycling of lithium-ion battery cathode material LiMn_2_O_4_. J. Mater. Chem. A **7**, 8845–8854 (2019). 10.1039/C8TA08083F

[74] T. Liu, A. Dai, J. Lu, Y. Yuan, Y. Xiao et al., Correlation between manganese dissolution and dynamic phase stability in spinel-based lithium-ion battery. Nat. Commun. **10**, 4721 (2019). 10.1038/s41467-019-12626-3 31624258 10.1038/s41467-019-12626-3 PMC6797712

[75] H. Yaghoobnejad Asl, A. Manthiram, Proton-induced disproportionation of jahn–teller-active transition-metal ions in oxides due to electronically driven lattice instability. J. Am. Chem. Soc. **142**, 21122–21130 (2020). 10.1021/jacs.0c10044 33284616 10.1021/jacs.0c10044

[76] W. Li, Review—an unpredictable hazard in lithium-ion batteries from transition metal ions: dissolution from cathodes, deposition on anodes and elimination strategies. J. Electrochem. Soc. **167**, 090514 (2020). 10.1149/1945-7111/ab847f

[77] Z. Zhao, W. Zhang, M. Liu, S.J. Yoo, N. Yue et al., Ultrafast nucleation reverses dissolution of transition metal ions for robust aqueous batteries. Nano Lett. **23**(11), 5307–5316 (2023). 10.1021/acs.nanolett.3c01435 37276017 10.1021/acs.nanolett.3c01435

[78] C. Zhan, T. Wu, J. Lu, K. Amine, Dissolution, migration, and deposition of transition metal ions in Li-ion batteries exemplified by Mn-based cathodes–a critical review. Energy Environ. Sci. **11**, 243–257 (2018). 10.1039/C7EE03122J

[79] L. Chen, W. Sun, K. Xu, Q. Dong, L. Zheng et al., How Prussian blue analogues can be stable in concentrated aqueous electrolytes. ACS Energy Lett. **7**, 1672–1678 (2022). 10.1021/acsenergylett.2c00292

[80] J. Agrisuelas, J.J. García-Jareño, D. Gimenez-Romero, F. Vicente, Insights on the mechanism of insoluble-to-soluble Prussian blue transformation. J. Electrochem. Soc. **156**, P149 (2009). 10.1149/1.3177258

[81] Y. Huang, S. Ren, Multifunctional Prussian blue analogue magnets: emerging opportunities. Appl. Mater. Today **22**, 100886 (2021). 10.1016/j.apmt.2020.100886

[82] C. Liu, X. Xie, B. Lu, J. Zhou, S. Liang, Electrolyte strategies toward better zinc-ion batteries. ACS Energy Lett. **6**, 1015–1033 (2021). 10.1021/acsenergylett.0c02684

[83] F. Wang, W. Sun, Z. Shadike, E. Hu, X. Ji et al., How water accelerates bivalent ion diffusion at the electrolyte/electrode interface. Angew. Chem. Int. Ed. **57**, 11978–11981 (2018). 10.1002/anie.201806748 10.1002/anie.201806748 30063278

[84] J. Yue, L. Lin, L. Jiang, Q. Zhang, Y. Tong et al., Interface concentrated-confinement suppressing cathode dissolution in water-in-salt electrolyte. Adv. Energy Mater. **10**, 2000665 (2020). 10.1002/aenm.202000665

[85] X. Dong, Y.-G. Wang, Y. Xia, Promoting rechargeable batteries operated at low temperature. Acc. Chem. Res. **54**, 3883–3894 (2021). 10.1021/acs.accounts.1c00420 34622652 10.1021/acs.accounts.1c00420

[86] H.Y. Asl, A. Manthiram, Reining in dissolved transition-metal ions. Science **369**, 140–141 (2020). 10.1126/science.abc5454 32646985 10.1126/science.abc5454

[87] J. Cattermull, M. Pasta, A.L. Goodwin, Structural complexity in Prussian blue analogues. Mater. Horiz. **8**, 3178–3186 (2021). 10.1039/d1mh01124c 34713885 10.1039/d1mh01124c PMC9326455

[88] Z. Caixiang, J. Hao, J. Zhou, X. Yu, B. Lu, Interlayer-engineering and surface-substituting manganese-based self-evolution for high-performance potassium cathode. Adv. Energy Mater. **13**, 2203126 (2023). 10.1002/aenm.202203126

[89] B. Liu, Q. Zhang, U. Ali, Y. Li, Y. Hao et al., Solid-solution reaction suppresses the Jahn-Teller effect of potassium manganese hexacyanoferrate in potassium-ion batteries. Chem. Sci. **13**, 10846–10855 (2022). 10.1039/d2sc03824b 36320692 10.1039/d2sc03824b PMC9491190

[90] F.D. Speck, A. Zagalskaya, V. Alexandrov, S. Cherevko, Periodicity in the electrochemical dissolution of transition metals. Angew. Chem. Int. Ed. **60**, 13343–13349 (2021). 10.1002/anie.202100337 10.1002/anie.202100337 PMC8252536 33687762

[91] T. Zhang, Y. Tang, S. Guo, X. Cao, A. Pan et al., Fundamentals and perspectives in developing zinc-ion battery electrolytes: a comprehensive review. Energy Environ. Sci. **13**, 4625–4665 (2020). 10.1039/D0EE02620D

[92] Z. Xing, G. Xu, J. Han, G. Chen, B. Lu et al., Facing the capacity fading of vanadium-based zinc-ion batteries. Trends Chem. **5**, 380–392 (2023). 10.1016/j.trechm.2023.02.008

[93] K. Nakamoto, R. Sakamoto, M. Ito, A. Kitajou, S. Okada, Effect of concentrated electrolyte on aqueous sodium-ion battery with sodium manganese hexacyanoferrate cathode. Electrochemistry **85**, 179–185 (2017). 10.5796/electrochemistry.85.179

[94] D.P. Leonard, Z. Wei, G. Chen, F. Du, X. Ji, Water-in-salt electrolyte for potassium-ion batteries. ACS Energy Lett. **3**, 373–374 (2018). 10.1021/acsenergylett.8b00009

[95] J. Han, H. Zhang, A. Varzi, S. Passerini, Fluorine-free water-in-salt electrolyte for green and low-cost aqueous sodium-ion batteries. ChemSusChem **11**, 3704–3707 (2018). 10.1002/cssc.201801930 30222910 10.1002/cssc.201801930

[96] L. Jiang, L. Liu, J. Yue, Q. Zhang, A. Zhou et al., High-voltage aqueous Na-ion battery enabled by inert-cation-assisted water-in-salt electrolyte. Adv. Mater. **32**, e1904427 (2020). 10.1002/adma.201904427 31782981 10.1002/adma.201904427

[97] Z. Hou, X. Zhang, X. Li, Y. Zhu, J. Liang et al., Surfactant widens the electrochemical window of an aqueous electrolyte for better rechargeable aqueous sodium/zinc battery. J. Mater. Chem. A **5**, 730–738 (2017). 10.1039/C6TA08736A

[98] Z. Liang, F. Tian, G. Yang, C. Wang, Enabling long-cycling aqueous sodium-ion batteries via Mn dissolution inhibition using sodium ferrocyanide electrolyte additive. Nat. Commun. **14**, 3591 (2023). 10.1038/s41467-023-39385-6 37328496 10.1038/s41467-023-39385-6 PMC10275921

[99] J. Chen, S. Lei, S. Zhang, C. Zhu, Q. Liu et al., Dilute aqueous hybrid electrolyte with regulated core-shell-solvation structure endows safe and low-cost potassium-ion energy storage devices. Adv. Funct. Mater. **33**, 2215027 (2023). 10.1002/adfm.202215027

[100] D. Zhang, L. Sun, C. Wang, Q. Xue, J. Feng et al., An open-framework structured material: [Ni(en)_2_]_3_[Fe(CN)_6_]_2_ as a cathode material for aqueous sodium- and potassium-ion batteries. ACS Appl. Mater. Interfaces **14**, 16197–16203 (2022). 10.1021/acsami.2c00143 35362955 10.1021/acsami.2c00143

[101] J. Chen, C. Liu, Z. Yu, J. Qu, C. Wang et al., High-energy-density aqueous sodium-ion batteries enabled by chromium hexacycnochromate anodes. Chem. Eng. J. **415**, 129003 (2021). 10.1016/j.cej.2021.129003

[102] J.-H. Lee, G. Ali, D.H. Kim, K.Y. Chung, Metal-organic framework cathodes based on a vanadium hexacyanoferrate Prussian blue analogue for high-performance aqueous rechargeable batteries. Adv. Energy Mater. **7**, 1601491 (2017). 10.1002/aenm.201601491

[103] J. Xie, L. Ma, J. Li, X. Yin, Z. Wen et al., Self-healing of Prussian blue analogues with electrochemically driven morphological rejuvenation. Adv. Mater. **34**, e2205625 (2022). 10.1002/adma.202205625 36114744 10.1002/adma.202205625

[104] U. Ali, B. Liu, H. Jia, Y. Li, Y. Li et al., In situ Fe-substituted hexacyanoferrate for high-performance aqueous potassium ion batteries. Small (2023). 10.1002/smll.202305866 10.1002/smll.202305866 37712131

[105] M.A. Oliver-Tolentino, J. Vázquez-Samperio, S.N. Arellano-Ahumada, A. Guzmán-Vargas, D. Ramírez-Rosales et al., Enhancement of stability by positive disruptive effect on Mn-Fe charge transfer in vacancy-free Mn-co hexacyanoferrate through a charge/discharge process in aqueous Na-ion batteries. J. Phys. Chem. C **122**, 20602–20610 (2018). 10.1021/acs.jpcc.8b05506

[106] Y. Ma, Y. Ma, S.L. Dreyer, Q. Wang, K. Wang et al., High-entropy metal–organic frameworks for highly reversible sodium storage. Adv. Mater. **33**, 2101342 (2021). 10.1002/adma.202101342 10.1002/adma.202101342 PMC11469271 34245051

[107] M. Du, P. Geng, C. Pei, X. Jiang, Y. Shan et al., High-entropy Prussian blue analogues and their oxide family as sulfur hosts for lithium-sulfur batteries. Angew. Chem. Int. Ed. **61**, e202209350 (2022). 10.1002/anie.202209350 10.1002/anie.202209350 36006780

[108] J. Xing, Y. Zhang, Y. Jin, Q. Jin, Active cation-integration high-entropy Prussian blue analogues cathodes for efficient Zn storage. Nano Res. **16**, 2486–2494 (2023). 10.1007/s12274-022-5020-0

[109] X. Zhao, Z. Xing, C. Huang, Investigation of high-entropy Prussian blue analog as cathode material for aqueous sodium-ion batteries. J. Mater. Chem. A **11**, 22835–22844 (2023). 10.1039/D3TA04349E

[110] B. Wenger, P.K. Nayak, X. Wen, S.V. Kesava, N.K. Noel et al., Consolidation of the optoelectronic properties of CH_3_NH_3_PbBr_3_ perovskite single crystals. Nat. Commun. **8**, 590 (2017). 10.1038/s41467-017-00567-8 28928482 10.1038/s41467-017-00567-8 PMC5605602

[111] M. Xue, Y. Wang, X. Wang, X. Huang, J. Ji, Single-crystal-conjugated polymers with extremely high electron sensitivity through template-assisted *in situ* polymerization. Adv. Mater. **27**, 5923–5929 (2015). 10.1002/adma.201502511 26308660 10.1002/adma.201502511

[112] H. Liang, X. Ma, Z. Yang, P. Wang, X. Zhang et al., Emergence of superconductivity in doped glassy-carbon. Carbon **99**, 585–590 (2016). 10.1016/j.carbon.2015.12.046

[113] D. Su, A. McDonagh, S.-Z. Qiao, G. Wang, High-capacity aqueous potassium-ion batteries for large-scale energy storage. Adv. Mater. **29**, 1604007 (2017). 10.1002/adma.201604007 10.1002/adma.201604007 27781313

[114] X. Wu, C. Wu, C. Wei, L. Hu, J. Qian et al., Highly crystallized Na_2_CoFe(CN)_6_ with suppressed lattice defects as superior cathode material for sodium-ion batteries. ACS Appl. Mater. Interfaces **8**, 5393–5399 (2016). 10.1021/acsami.5b12620 26849278 10.1021/acsami.5b12620

[115] D. Cai, X. Yang, B. Qu, T. Wang, Comparison of the electrochemical performance of iron hexacyanoferrate with high and low quality as cathode materials for aqueous sodium-ion batteries. Chem. Commun. **53**, 6780–6783 (2017). 10.1039/C7CC02516E 10.1039/c7cc02516e 28597877

[116] C. Li, X. Wang, W. Deng, C. Liu, J. Chen et al., Size engineering and crystallinity control enable high-capacity aqueous potassium-ion storage of Prussian white analogues. ChemElectroChem **5**, 3887–3892 (2018). 10.1002/celc.201801277

[117] W. Zhang, L. Xia, C. Shi, R. Qi, M. Hu, Casting and recycling of insoluble, labile single-crystal coordination polymer through reversible solid-liquid-solid transition. Matter **6**, 3394–3412 (2023). 10.1016/j.matt.2023.05.024

[118] X. Liu, Y. Cao, J. Sun, Defect engineering in Prussian blue analogs for high-performance sodium-ion batteries. Adv. Energy Mater. **12**, 2202532 (2022). 10.1002/aenm.202202532

[119] M. Wan, R. Zeng, J. Meng, Z. Cheng, W. Chen et al., Post-synthetic and *in situ* vacancy repairing of iron hexacyanoferrate toward highly stable cathodes for sodium-ion batteries. Nano-Micro Lett. **14**, 9 (2021). 10.1007/s40820-021-00742-z 10.1007/s40820-021-00742-z PMC8642478 34862572

[120] F. Peng, L. Yu, S. Yuan, X.-Z. Liao, J. Wen et al., Enhanced electrochemical performance of sodium manganese ferrocyanide by Na_3_(VOPO_4_)_2_F coating for sodium-ion batteries. ACS Appl. Mater. Interfaces **11**, 37685–37692 (2019). 10.1021/acsami.9b12041 31525888 10.1021/acsami.9b12041

[121] F. Feng, S. Chen, S. Zhao, W. Zhang, Y. Miao et al., Enhanced electrochemical performance of MnFe@NiFe Prussian blue analogue benefited from the inhibition of Mn ions dissolution for sodium-ion batteries. Chem. Eng. J. **411**, 128518 (2021). 10.1016/j.cej.2021.128518

[122] C. Xu, Y. Ma, J. Zhao, P. Zhang, Z. Chen et al., Surface engineering stabilizes rhombohedral sodium manganese hexacyanoferrates for high-energy Na-ion batteries. Angew. Chem. Int. Ed. **62**, e202217761 (2023). 10.1002/anie.202217761 10.1002/anie.202217761 36719001

[123] M. Lucero, D.B. Armitage, X. Yang, S.K. Sandstrom, M. Lyons et al., Ball milling-enabled Fe^2.4^+ to Fe^3^+ redox reaction in Prussian blue materials for long-life aqueous sodium-ion batteries. ACS Appl. Mater. Interfaces **15**, 36366–36372 (2023). 10.1021/acsami.3c07304 37481736 10.1021/acsami.3c07304

[124] E. Nossol, V.H.R. Souza, A.J.G. Zarbin, Carbon nanotube/Prussian blue thin films as cathodes for flexible, transparent and ITO-free potassium secondary battery. J. Colloid Interface Sci. **478**, 107–116 (2016). 10.1016/j.jcis.2016.05.056 27288576 10.1016/j.jcis.2016.05.056

[125] M. Morant-Giner, R. Sanchis-Gual, J. Romero, A. Alberola, L. García-Cruz et al., Prussian Blue@MoS_2_ layer composites as highly efficient cathodes for sodium-and potassium-ion batteries. Adv. Funct. Mater. **28**, 1706125 (2018). 10.1002/adfm.201706125

[126] M. Zhang, T. Dong, D. Li, K. Wang, X. Wei et al., High-performance aqueous sodium-ion battery based on graphene-doped Na_2_MnFe(CN)_6_–zinc with a highly stable discharge platform and wide electrochemical stability. Energy Fuels **35**, 10860–10868 (2021). 10.1021/acs.energyfuels.1c01095

[127] C.D. Wessells, S.V. Peddada, R.A. Huggins, Y. Cui, Nickel hexacyanoferrate nanoparticle electrodes for aqueous sodium and potassium ion batteries. Nano Lett. **11**, 5421–5425 (2011). 10.1021/nl203193q 22043814 10.1021/nl203193q

[128] Y. Zhang, J. Xu, Z. Li, Y. Wang, S. Wang et al., All-climate aqueous Na-ion batteries using “water-in-salt” electrolyte. Sci. Bull. **67**, 161–170 (2022). 10.1016/j.scib.2021.08.010 10.1016/j.scib.2021.08.010 36546009

[129] T.Y. Pan, C.Y. Ruqia, C.S. Wu, S.G. Ni et al., Improvement in cycling stability of Prussian blue analog-based aqueous sodium-ion batteries by ligand substitution and electrolyte optimization. Electrochim. Acta **427**, 140778 (2022). 10.1016/j.electacta.2022.140778

[130] J. Liu, C. Yang, B. Wen, B. Li, Y. Liu, Ultra-long cycle of Prussian blue analogs achieved by equilibrium electrolyte for aqueous sodium-ion batteries. Small **19**, e2303896 (2023). 10.1002/smll.202303896 37460403 10.1002/smll.202303896

[131] S. Husmann, A.J.G. Zarbin, R.A.W. Dryfe, High-performance aqueous rechargeable potassium batteries prepared via interfacial synthesis of a Prussian blue-carbon nanotube composite. Electrochim. Acta **349**, 136243 (2020). 10.1016/j.electacta.2020.136243

